# Integrating Tumor Hypoxic Sensing and Photothermal Therapy Using a Miniaturized Fiber‐Optic Theranostic Probe

**DOI:** 10.1002/smsc.202400450

**Published:** 2024-10-31

**Authors:** Fangzhou Jin, Zhiyuan Xu, Wei Wang, Zhongyuan Cheng, Yang Wu, Zesen Li, Enlai Song, Xu Yue, Yong Kang Zhang, Wei Li, Youzhen Feng, Donglin Cao, Dongmei Zhang, Minfeng Chen, Xiangran Cai, Yang Ran, Bai‐Ou Guan

**Affiliations:** ^1^ Guangdong Provincial Key Laboratory of Optical Fiber Sensing and Communications, Institute of Photonics Technology Jinan University Guangzhou 510632 China; ^2^ College of Physics & Optoelectronic Engineering Jinan University Guangzhou 510632 China; ^3^ Department of Laboratory Medicine The Affiliated Guangdong Second Provincial General Hospital of Jinan University Guangzhou 510317 China; ^4^ Medical Imaging Center The First Afﬁliated Hospital of Jinan University Guangzhou 510630 China; ^5^ State Key Laboratory of Bioactive Molecules and Druggability Assessment Jinan University Guangzhou 510632 China

**Keywords:** imaging‐assisted navigation, microenvironment sensing, optical fibers, photothermal therapy, tumor theranostics

## Abstract

Efficient delivery of photons to visceral organs is critical for the treatment of deep‐seated tumors taking advantage of photo theranostics. Optical fiber can be regarded as a direct and facile photon pathway for targeting tumor lesion. However, current fiber theranostic strategies rely on the spatially separated optical fibers to realize diagnosis and therapy independently, resulting in low compactness, poor continuity of medical process, and incompatibility with current medical technologies. Herein, an integrated fiber‐optic theranostic (iFOT) probe is developed that merges tumor microenvironment sensing and photothermal therapy by functionalizing the fiber with graphene/gold nanostar hybrid materials and hypoxic‐responsive fluorophores. The iFOT probe can quickly detect the hypoxia of xenograft tumors of mice with high sensitivity. The tumors can be photothermally killed on‐site through the same fiber probe tightly followed by detection, which presents a high cure rate. More importantly, the iFOT is highly adaptable to the conventional medical imaging and endoscopic techniques, which facilitates the imaging‐assisted navigation and manipulation by use of the interventional trocar. The proposed strategy can be used as an effective endoscopic and interventional tool for tackling deep‐situated tumor and may open a revolutionized pathway to bridge separate diagnosis and therapy process in the current stage.

## Graphical abstract

Herein, a fiber optic probe integrating the tumor sensing and photothermal therapy functionalities is proposed to tackle solid tumors. The strategy can achieve spatial integration and functional integration in a single‐fiber probe that can perform diagnosis and cancer treatment. Moreover, it has high compatibility with minimally invasive interventional surgery and medical imaging techniques in clinical medicine.

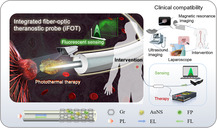

## 1 Introduction

Cancer has been one of the most serious public health threats to human beings worldwide. There were 19.3 million new cancer cases in 2020, and the number is estimated to 28.4 million in 2040. Conventional tumor medicine feels increasingly powerless as it confronts the surging and diverse demands of cancer diagnosis and therapy.^[^ ^1^ ^]^ Compared with traditional therapeutic approaches, such as the typical “open” tumorectomy, endoscopic or interventional tumor therapy garners more credits toward deep‐seated lesion due to its minimal invasive manner, time efficiency, and negligible side effect. In this realm, optical fiber, which has the advantages of electric isolation, resistance to corrosion, hair‐like miniature size, maneuverability, and versatility, can be perfectly niched into the endoscopic and interventional tumor diagnosis and treatments.^[^ ^2^ ^]^

To date, optical fiber has been leveraged as the real‐time fiber endoscope,^[^ ^3^ ^]^ tumor microenvironment (TME) sensor,^[^ ^4^ ^]^ and tumor marker sensitive fluorescent fiber sensor^[^ ^5^ ^]^ for cancer diagnosis. Furthermore, the fiber‐based laser ablation,^[^ ^6^ ^]^ fiber‐assisted phototherapy,^[^ ^7^ ^]^ fiber‐optic theranostic needle,^[^ ^8^ ^]^ and optical microfiber‐based photonic hyperthermia needle^[^ ^9^ ^]^ were also developed to realize cancer treatment. Despite with significant progress, the current fiber‐optic tumor medicine strategy remains in its infancy toward clinical application. One major hurdle lies in the spatially fragmented sensing and actuation fiber elements, which separate the diagnosis and therapy processes, leading to discontinuity of medical care, larger footprint and elaborated configuration of sensor head, and failure to match current clinical requirement.^[^ ^8^ ^]^ Fortunately, exploiting the potential functionalities and then realizing functional integration are an eternal impetus for fiber‐optics to revolutionize the world.^[^ ^10^ ^]^ Laying on seabed, a fiber cable can distributedly monitor the seafloor earthquakes and tsunamis while fulfilling the duty of data transmission.^[^ ^11^ ^]^ Deployed into brain, optogenetics orchestrates the optical stimulation and electrophysiological readout using a single‐fiber probe.^[^ ^12^ ^]^ Likewise, the integration of diagnostic and therapeutic capabilities in a unified optical fiber not only sticks to the development path of optical fiber technology, but also enables practical implications. For example, interventional surgery, which features the negligible invasion, harnesses a microcatheter or a trocar to navigate and establish channel inside the body for the tumor sampling and treatment. The microcatheter has a smallest inner diameter below 0.5 mm,^[^ ^13^ ^]^ necessitating extreme compactness of the fiber‐optic probe. Furthermore, the troublesome and time‐consuming diagnosis and treatment procedure can be greatly simplified through one “plug and play” theranostic mode. Finally, the abundant optical spectral reservoir allows the simultaneous multiple operations for enabling the intraoperative tumor monitoring.

**Figure 1:**
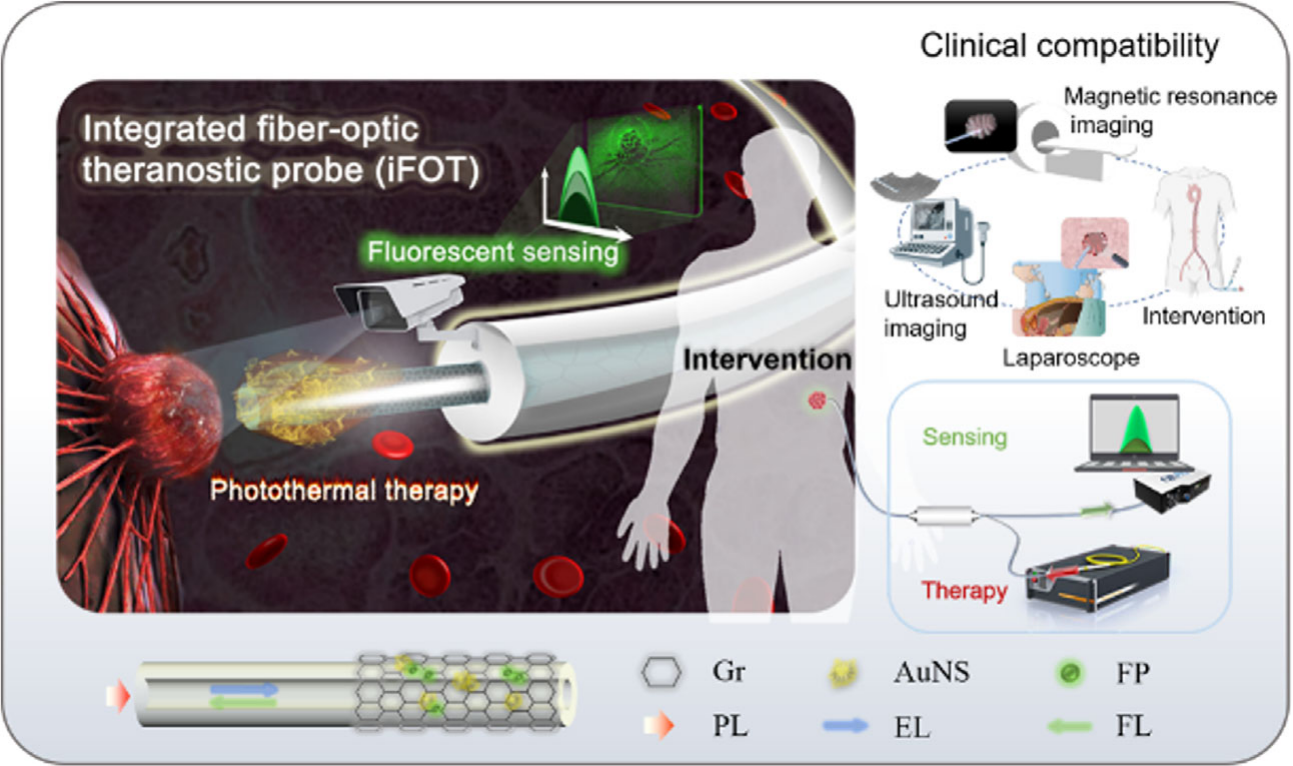
Diagram of imaging‐compatible iFOT probe for tumor sensing and therapy. The construction of multifunctional fiber probe that integrates in vivo tumor detection and PTT. Nitroreductase (NTR) probes on fiber can fluorescently identify tumors through the interaction with the hypoxia markers and Gr/AuNS nanomaterials implement the photothermal conversion while boosting the fluorescent signal. In addition, the iFOT probe is compatible with multiple medical techniques, such as laparoscope, US imaging, MRI, and interventional surgery, which can facilitate the iFOT probe navigation inside the human body. Gr, graphene; AuNS, gold nanostar; FP, fluorescent probe; PL, pump laser; EL, excitation light; FL, fluorescent light.

Herein, we proposed a fiber‐optic probe integrating the TME sensing and photothermal therapy (PTT) functionalities to tackle solid tumors. At first, the optimization between the evanescent field utilization and structural rigidity was explored in the design of fiber probe, which might be otherwise a challenge for the functional integration and in vivo manipulation.^[^ ^9^, ^14^ ^]^ In this design, hypoxia‐indicated fluorescent reagents and graphene/gold nanostar (Gr/AuNS) hybrid nanomaterials were immobilized at the fiber surface to achieve a combined strategy of tumor hypoxia microenvironment detection and PTT in vivo. Graphene and AuNS are essential tools for endowing the bare optical fiber with efficient photothermal and sensitive sensing capabilities. Due to the excellent photothermal effect of graphene,^[^ ^15^ ^]^ it can perform as a photothermal converter, absorbing low‐power pump laser light via fiber evanescent field and release a substantial amount of thermal energy to realize tumor PTT. The hypoxia‐responsive fluorescent indicators were functionalized on the graphene film to realize TME sensing. The AuNS hybridized in the graphene film pronouncedly enhanced the intensity of electromagnetic (EM) field,^[^ ^16^ ^]^ which can surmount the fluorescent quenching arising from graphene absorption,^[^ ^17^ ^]^ thereby further enhancing the fiber sensing capability. Additionally, the plasmonic effect of AuNS can also promote the photothermal conversion of graphene, further enhancing the fiber PTT.^[^ ^18^ ^]^ The integrated fiber‐optic theranostic (iFOT) probe can complete the in vivo detection of hypoxia in tumor within 10 s. Furthermore, PTT efficacy on the tumor‐bearing mice was also greatly improved due to the high photothermal conversion of the hybrid nanomaterials on the fiber. More importantly, to pave the way for clinical translation, the endoscopic and interventional treatment capability of iFOT probe was demonstrated. In the demonstration, the iFOT probe could be precisely observed and manipulated under the laparoscope, magnetic resonance imaging (MRI), medical ultrasound (US) imaging, and photoacoustic imaging (PAI), manifesting its compatibility with the conventional medical imaging techniques. Furthermore, dual modal sensing, monitoring, and therapeutic evaluation were achieved thanks to the merging of iFOT and MRI imaging technology. The results shed light on the potential of imaging‐assisted fiber internal navigation, synergistic diagnosis, and TME monitoring after therapy, and thus lay the foundation for the clinical translation (**Figure** **1**).

## 2 Results

**Figure 2:**
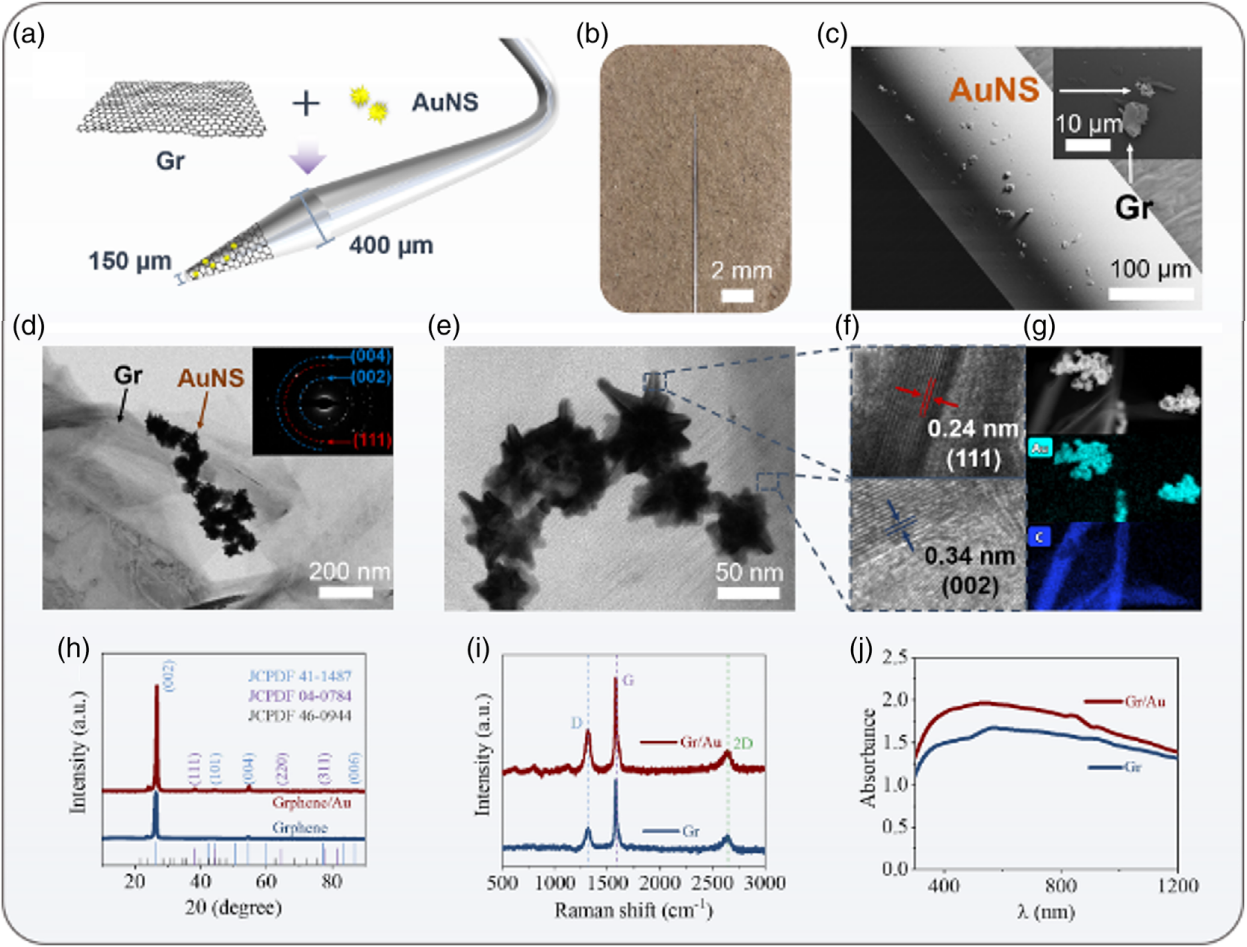
The characterization of Gr/AuNS on fiber. a) Scheme of the preparation procedures of the Gr/AuNS fiber probe. b) Photography of the fiber tip. c) SEM images of the Gr/AuNS on fiber. d,e) TEM images of the Gr/AuNS nanostructure (the inset in Figure 2d is the corresponding). f) High‐resolution TEM images of the Gr/AuNS. g) Elemental mapping images of the Gr/AuNS nanostructure. h) XRD patterns, i) Raman spectra, and j) UV–vis spectra of the pure graphene and the Gr/AuNS.

The multifunctional fiber probe was realized by immobilization of Gr/AuNS hybrid nanomaterials on the fiber taper (**Figure** **2**a). At first, graphene was activated for the introduction of reactive functional groups.^[^ ^19^ ^]^ To endow activated graphene with enhanced photothermal conversion efficiency and address the fluorescent quenching mediated by graphene, Gr/AuNS suspensions were prepared by mixing the synthesized AuNS with the activated graphene. To integrate Gr/AuNS onto the optical fiber, the procedures of hydroxylation, silanization, and immobilization are followed sequentially as reported previously.^[^ ^5^, ^8^ ^]^ Activated graphene with enriched functional groups and AuNS can both strongly interact with silane through hydrogen bonding and electrostatic adsorption, and thus generate stable Gr/AuNS composite on the fiber.^[^ ^20^ ^]^ In the following, the NTR fluorescent molecules, which can emit green fluorescence via blue light excitation, were also tethered to the fiber by the covalent bonds. The tailored AuNS (S10) with the plasmonic peak of 550 nm can be orchestrated with the fluorescent emission, heralding the enhancement of fluorescent signal.

Figure 2b shows the preparation procedure of the iFOT and the microscopic photograph of the fiber tip. The cone structure of the fiber tip cannot only facilitate the tissue intervention, but also enhance the interaction between the delivered light and NTR‐activated fluorophore via evanescent field. The configuration of Gr/AuNS‐decorated fiber (Gr/AuNS fiber) sensor was confirmed by scanning electron microscopy (SEM) and transmission electron microscopy (TEM) (Figure 2 and S1, Supporting Information). As shown in Figure 2c, Gr/AuNS were dispersedly attached on the surface of the 150 μm fiber tip, providing the basis for enhanced sensing and photothermal conversion. 2D graphene sheets with surface diameters ranging from 2 to 9 μm were observed to provide favorable anchor sites for AuNS, which were randomly distributed on and around the graphene sheets. This result was further confirmed by TEM images (Figure 2d). As shown in Figure 2e, AuNS with an average aspect ratio of 70 nm could be observed on graphene. Compared to gold nanospheres, the engineered AuNS possessed the emission‐like branches that can drastically enhance the local EM field to boost fluorescent signal. The selected area electron diffraction pattern displays two sets of diffraction rings that can be indexed to the graphic structure (blue rings) and the gold structure (red rings). It corresponds to the two kinds of lattice fringes shown in high‐resolution TEM (Figure 2f). The lattice spacing of about 0.34 and 0.24 nm corresponds to the (002) planes of the hexagonal graphite structure of graphene and the (111) plane of cubic Au, respectively. Figure 2g demonstrates the elemental mapping images also confirmed the distribution of graphene and AuNS directly.

Figure 2h shows the X‐ray diffractometer (XRD) patterns of the pure graphene and the Gr/AuNS with different interfaces. The diffraction peaks of Gr/AuNS in the XRD pattern matched well with those of graphene (JCPDS no. 41‐1487) and Au (JCPDS no. 04‐0784), which suggested the successful combination of graphene and AuNS. Raman spectra of the Gr/AuNS exhibited D‐band at 1315 cm^−1^ and G‐band at 1580 cm^−1^ (Figure 2i), respectively, which is the typical characteristic of graphene. Due to the sensitization mechanism of AuNS, the Raman spectral signal of Gr/AuNS is remarkably higher than that of graphene. Moreover, the UV–vis spectra Figure 2j showed that the Gr/AuNS had a broad‐spectrum absorption and relatively higher absorption than pure graphene in the near‐infrared light window, indicating that the hybridized AuNS could boost both the fluorescence intensity and photothermal conversion to the pure graphene.

**Figure 3:**
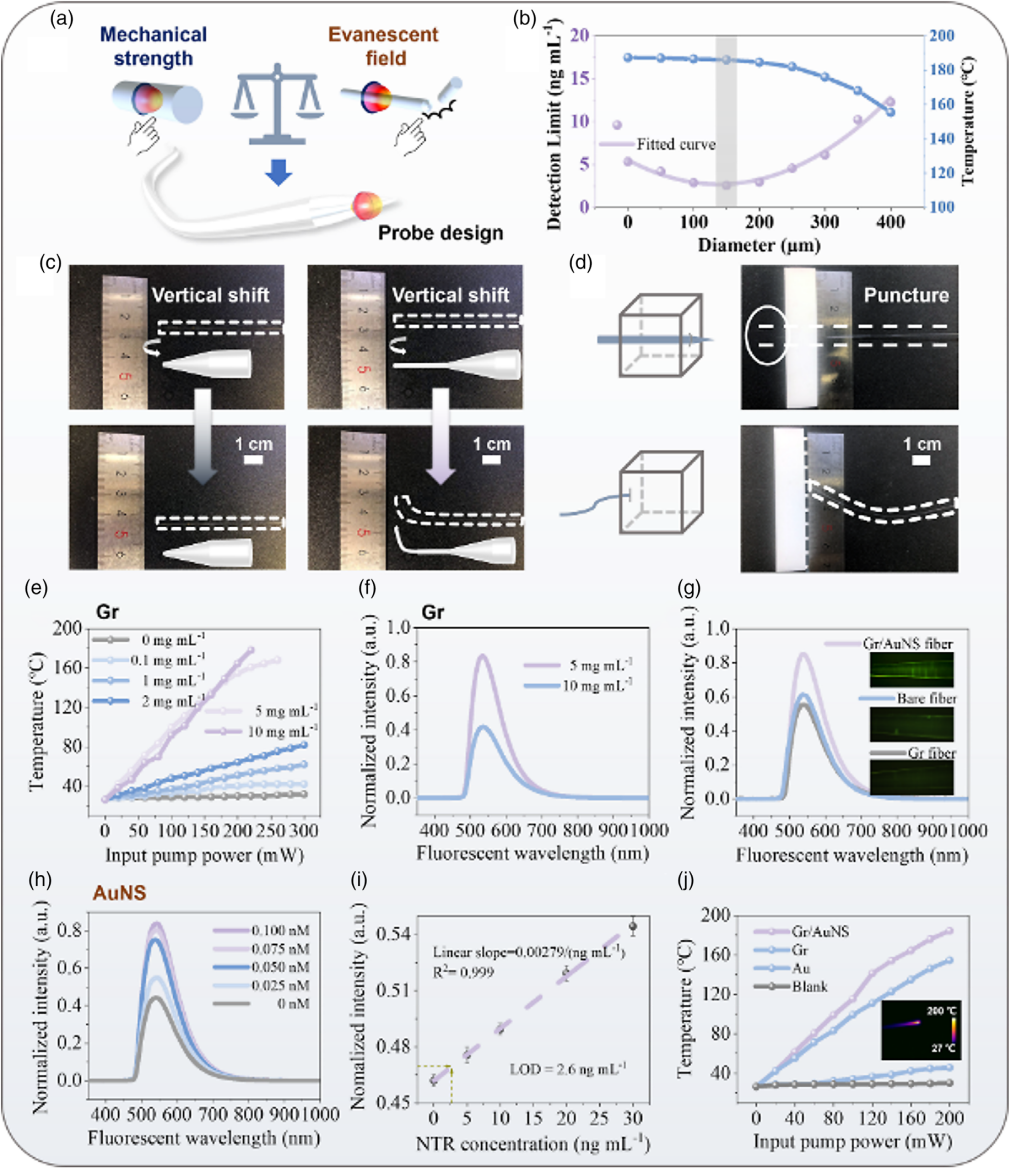
Optimization of fiber‐optic probe. a) Scheme of the fiber probe design for optimizing fluorescence and photothermal effect. b) LOD and temperature regarding the optical fibers with different end diameters. c) Deformation of different fibers after vertical shifts. d) Rigidity of different fiber head structures in the puncture operation. e) The influence of graphene content on the photothermal conversion of the Gr/AuNS fiber. f) The influence of high graphene content for fiber fluorescence sensing. g) Sensing performance and calibration of the optical fiber probe (blank, pure graphene, and Gr/AuNS) for detecting NTR in vitro. Even though the fluorescence quenching effect of graphene decreases the fluorescence intensity, the downside is effectively improved by the combination strategy of graphene and AuNS. h) The influence of AuNS concentration on fluorescence sensing. The fluorescent signal went up as the increase of AuNS content. i) The ≈550 nm fluorescent signal of Gr/AuNS fiber increases with the increment of the NTR density. For a smaller detection range (0–30 ng mL^−1^), the curve could be approximately fitted in a linear regression function, *I* = 0.00278 × *C* + 0.46207 (*R* ^2^ = 0.983) (*I*: intensity; *C*: concentration of NTR). The LOD of the sensor can be obtained to ≈2.6 ng mL^−1^ and the LOQ of the sensor can be obtained to ≈8.5 ng mL^−1^, according to the equation of X_LOD_ = *f* ^−1^ ($\bar{y_{\text{blank}}}$ + 10/3 × *σ*) and X_LOQ_ = *f* ^−1^ ($\bar{y_{\text{blank}}}$ + 10 × *σ*); (*f*: function; $\bar{y_{\text{blank}}}$ : mean value of the blank sample tests; *σ*: standard deviation). Error bars are obtained by three dependent measurements. j) Photon‐induced temperature changes in the air using bare fiber, graphene fiber, AuNS fiber, and Gr/AuNS fiber as a function of the pump power. (Inset: the IR record of the temperature of Gr/AuNS fiber under the pump power of 160 mW).

To achieve fluorescence‐based detection and photothermal actuation simultaneously with the same fiber probe, we designed the fiber probe by customizing the diameter of the fiber end and modulating the decorated nanomaterials as shown in **Figure** **3**a. The light–mater interaction with regard to the fluorescent molecules and photosensitizers on the fiber surface can be directly influenced by the thickness of fiber probes, which provide different intensity of evanescent fields. For another, the structural morphology also determines the mechanical strength of fiber probe. Therefore, it is important to balance the evanescent field utilization and rigidity of fiber probe. Furthermore, the interference between fluorescent agents and photosensitizers on a fiber probe needs a deeper understanding to strengthen both effects.

The evanescent field utilization, which can be represented by the photothermal effect and fluorescent intensity, related to the diameter of fiber probe is described in Figure 3b. As the diameter of fiber‐end decreases, the photothermal conversion efficiency is enhanced, but the efficiency curve presents a flat region as the diameter is less than 150 μm due to the evanescent field is already taken fully advantage of below that diameter, enabling a presence of ≈186 °C in the air under the incidence of 250 mW pump light. By contrast, the fluorescent sensing curve has a distinct style. The fiber probe has the lowest limit‐of‐detection (LOD) of 2.6 ng mL^−1^ in the calibration test when the fiber end diameter is designed at 150 μm (Figure S2 and S3, Supporting Information). This parabolic‐like curve implies there should be a compromise between the intensity of evanescent field and the amount of fluorophore loading regarding the thickness of fiber. Therefore, the fiber with an end diameter of 150 μm was selected as the optimal probe to realize the integrated theranostics.

More importantly, the optimized structure of fiber probe can also ensure its mechanical strength, which is suitable for realizing in vivo test. Two types of fiber end with different diameters (*D* = 150 μm/TL = 5 mm and *D* = 40 μm/*TL* = 10 mm; *D*: diameter, *TL*: transition length) were fabricated to improve the understanding on the relationship between the mechanical strength and the structure of fiber. In Figure 3c, after a 2 centimeter of lateral translation, the thicker fiber probe (which is preferred to fabricate iFOT) maintained its straightness, while the thinner microfiber exhibited bending. Subsequently, to evaluate the feasibility of in vivo operations of the fiber probe, we further tested the rigidity of the two fiber probes through puncturing them into the soft rubber. As shown in Figure 3d, the optimized fiber probe (150 μm) can easily penetrate through the white rubber, but the microfiber was broken during the piercing process, and the original fiber (*D* = 400 μm) also failed to stab into the rubber (Figure S4, Supporting Information). The results reveal that the optimized needle‐like head structure not only allows a high pressure exerting on the contact surface to realize paracentesis, but also yields the pronounced counter‐bending ability—a key feature for the clinical operation, especially the interventional surgery, which relies on the catheter and puncture needle to reach the lesion. A highly bendable head structure will undermine the compatibility of the probe to the interventional surgery due to the lack of controllability in the rigid needle and silky ductility in the soft catheter. The rigidity of the optimized probe head derives from the larger area inertia moment and sectional resistance moment provided by the gradually diameter changed (cone) structure (Figure S5 and S6, Supporting Information).

For tumor PTT, the temperature elevation of the probe is prioritized. Graphene is a perfect photothermal absorber and its quantity of decoration is crucial to determine the photothermal efficiency. As the graphene quantity of decoration increases, represented by the density of the graphene suspension in the decoration, the iFOT can reach higher temperatures under the same laser power stimulation. As shown in Figure 3e, the fiber probe using graphene suspension of 5 mgmL^−1^reached 155 °C, showing a significant temperature elevation compared with the suspension of lower density. However, further increasing the density of graphene, for example, to 10 mg mL^−1^ did not bring about a greater enhancement of photoheating outcome due to the limited capacity of surface area of the fiber end, but would severely quench the fluorescent intensity for TME sensing (Figure 3f).

AuNS was then introduced to mitigate the fluorescent quenching resulted from the presence of graphene and potentiate the fluorescence detection efficiency. As shown in Figure 3g, the bare silica fiber, Gr only fiber, and Gr/AuNS fiber were employed to make comparison regarding the fluorescent sensing performance in the PBS‐NTR‐fluorescent molecule mixture solution. Due to the fluorescent quenching effect of graphene, the fluorescence signal intensity collected by the Gr fiber sensor was weaker than the bare silica fiber. Taking advantage of the EM enhancement effect of the AuNS, the Gr/AuNS fiber could not just overcome the graphene induced deterioration of fluorescent signal but even enable a 1.5 times higher fluorescent intensity compared with the bare silica fiber. The fluorescence intensity of the fiber probe samples was in agreement with the photographs under of fluorescence microscopy. The effect of the morphology of gold nanostructures on the intensity of the fluorescence signal was analyzed and customized to obtain the high fluorescence signal (Figure S7, Supporting Information). Like the photothermal characterization using graphene, the fluorescence signal can as well enhance by increasing concentration of AuNS colloidal solution and the saturation concentration regarding the fluorescent intensity enhancement was 0.1 nM, as shown in Figure 3h. The reason can also be attributed to the limitation of space for anchoring AuNS.

To characterize the fluorescence sensing properties of iFOT probe, we conducted in vitro calibration by inserting the fiber‐tip probes into PBS‐NTR‐fluorescent molecule mixtures with different concentrations of NTR (Figure 3i). The normalized intensity of the emission peak enhanced with the increase of NTR concentration (Figure S8, Supporting Information). For the wide NTR concentration range in Figure S9 (Supporting Information), the measured points of the peak intensity ratio can be well depicted by logistic fitting (*R* ^2^ = 0.989). And at the range of lower concentrations ranging from 0 to 30 ng mL^−1^, an approximately linear correlation was deduced, presenting the LOD of 2.6 ng mL^−1^ and limit of quantification (LOQ) of 8.5 ng mL^−1^. The long‐term stability and high‐temperature resistance of iFOT were also verified, as shown in Figure S10 (Supporting Information).

Surprisingly, AuNS not only enhanced fluorescence, but also further upgraded the photothermal efficiency of the fiber probe. Figure 3j shows the temperature rising of different fiber probes in the air under laser pumping. It can be seen that both Gr and Gr/AuNS fiber can achieve high local temperatures (>100 °C) and the presence of AuNS provides an additional elevation of local temperature due to the gold plasmonic effect^[^ ^21^ ^]^ (Figure S11, Supporting Information). Combined with the above description, we achieved the optimization strategy of the iFOT probe, which is important for the implementation of fluorescence detection and PTT in vivo.

**Figure 4:**
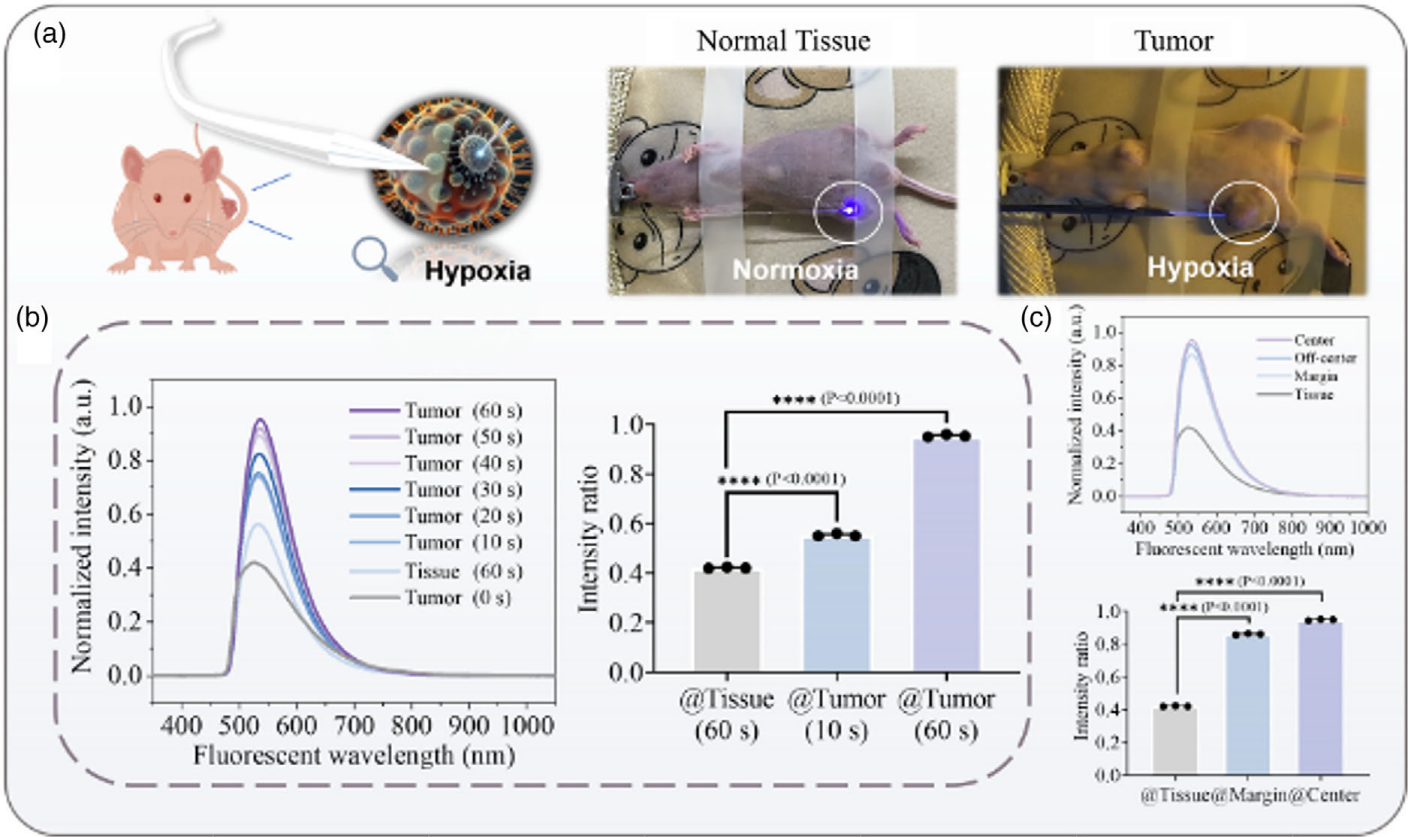
Fluorescent sensing of iFOT for in situ tumor detection. a) Schematic diagram of in vivo fluorescence detection in tumor‐bearing mice and healthy mice. b) The fiber sensing was harnessed to intervene in the normal tissue and MDA‐MB‐231 tumor in the mirror spots, respectively. The intratumor manipulation was shown in the real experiment image and video S1 (Supporting Information). Other than the normal tissue that brings about little effective signal, the tumor allows the fluorescent signal accumulation with respect to time. The curve originates from the normal tissue providing a baseline for the quantitative analysis of the tumor‐associated fluorescent regarding the detection time. Even a 10 s test for tumors showed great significance in comparison with normal tissue, manifesting the fast and real‐time determination capability. ****P < 0.0001. c) The fiber sensing was harnessed to intervene in the different sites (center, off‐center, and margin) of the same MDA‐MB‐231 tumor, respectively. Fluorescent signal evolution with time accumulation at the center, off‐center, and margin of the tumor. Signal intensity is proportional to tumor density. ****P < 0.0001.

Since the local endogenous hypoxia is a significant characteristic for the TME, the hypoxia‐associated biomarker, NTR, was selected as the tumor‐associated marker.^[^ ^22^ ^]^ The distinction between normal tissue and solid tumors could be evaluated by the presence of the NTR. NTR fluorescent molecules were immobilized on the fiber tip using the chemical bonding method.^[^ ^5^, ^8^ ^]^ The sensing principle of the iFOT probe for tumor diagnosis is shown in **Figure** **4**a. The presence of NTR results in the reduction reaction of fluorescent molecules, which would then emit strong green fluorescence at 550 nm, as they were excited by 450 nm blue light. To characterize the fluorescence sensing properties of iFOT probe, we injected the PBS‐NTR‐fluorescent molecule mixtures into pork tissue and used iFOT to detect NTR in situ. The result shows that a significantly positive response can be obtained in contrast with the test on the blank sample (Figure S12, Supporting Information).

Further, we conducted in vivo experiments using tumor‐bearing mice to demonstrate the feasibility of iFOT for solid tumor detection. As shown in Figure 4b, the tumor tissues in mice bearing orthotopic breast cancer and the corresponding sites of normal mice were punctured and detected through the iFOT probe, separately. For tumor detection, the fluorescent signal responds rapidly and offers a positive peak in 10 s. With the accumulation of NTR fluorescent molecules and NTR reactions, the signal intensity enhanced 1.7 times within 1 min and the wavelength was redshifted up to 5.27 nm on the spectrum. Conversely, no obvious fluorescence signal was detected in normal mouse tissues tested by focused fluorescence for 1 min in the same way. Compared to the normal tissue, the tumor trial showed positive results within the same detection time. Notably, the different fluorescent signals were also observed at different sites of the same tumor (Figure 4c and Figure S13, Supporting Information). The intensity of fluorescence signal was proportional to the tumor density. The results indicated that the fluorescence signal was highest in the central region of tumor while was lowest at the tumor edge, which was significantly variable compared with normal tissue. Moreover, iFOT can evaluate NTR levels in vivo after treatment (Figure S14, Supporting Information). Furthermore, the brief structure and rapid response of iFOT are visually displayed in video S4 (Supporting Information). Taken together, these results showed the iFOT can be used for rapid solid tumor detection in parallel with an effective tool for tumor margin detection. It is promising as an effective tool for intraoperative precise tumor localization.

**Figure 5:**
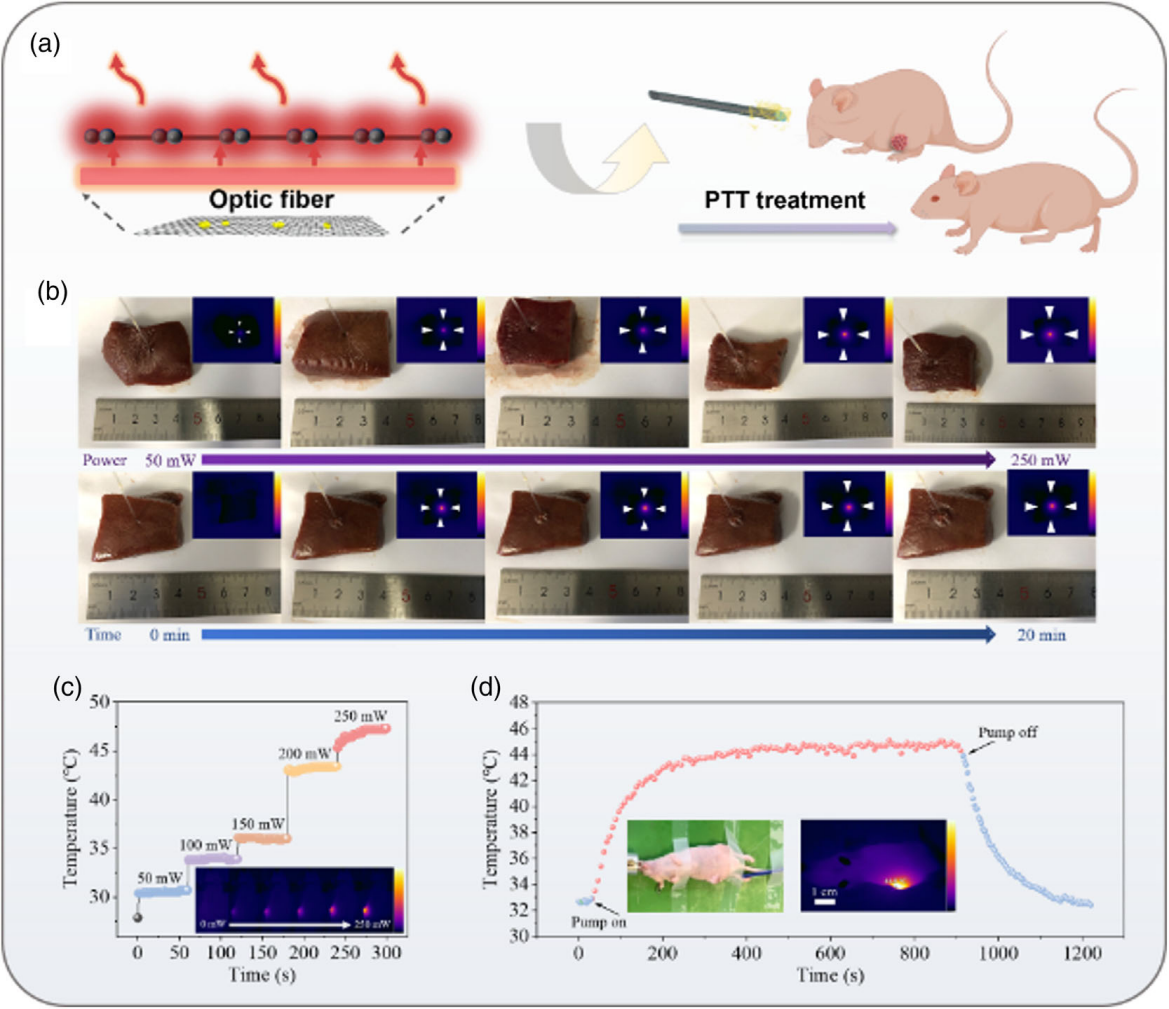
Tumor characterization and anticancer efficacy of the Gr/AuNS PTT fiber. a) Schematic diagram of photothermal conversion of iFOT and PTT of tumor in mice. b) Testing and characterization of the variation of the fiber thermal actuation range with pump power and time in vitro tissue. c) Temperature increment dictated by the step‐changed pump power. The inset image: the IR images that reveal the thermalization with the increment of pump power. d) Sensorgram of the photoheating in a demo of treatment in vivo. The entire process was recorded in Video S2, Supporting Information.

Next, we investigated the solid tumor PTT capability of the same iFOT probe. As shown in **Figure** **5**a, taking advantage of the excellent photothermal conversion efficiency of graphene, the graphene decorated fiber can release a large amount of heat in the surrounding area under 980 nm pump laser excitation, which is essential for tumor PTT.

Then, the photothermal imager was used to characterize the thermal radiation range of Gr/AuNS fiber tip to verify the effectiveness of fiber PTT and the action interval of PTT in vitro pig liver tissues (Figure 5b). The range of fiber thermal radiation was proportional to the power of the pump laser. After 5 min laser radiation of 200 mW, the photothermal capacity reached the peak, and the diameter and average temperature of the heated region were 9.9 mm and 42.6 °C, respectively. Compared to the previous reported PTT fiber,^[^ ^8^ ^]^ Gr/AuNS fiber performs a higher efficiency of photothermal conversion. (Figure S15, Supporting Information). In addition, iFOT probe also has good biosafety thanks to the low‐power laser and nonresidual nanoparticles (Figure S16, Supporting Information).

To verify the PTT efficacy for tumor treatment, we conducted the iFOT therapy experiment in vivo. For characterizing the PTT effect in vivo, the photothermal imager and an additional optical fiber Bragg grating sensor were used to monitor the temperature of the lesion area. As shown in Figure 5c, the tumor temperature was quantified in the pump power range of 0–250 mW, and the tumor temperature increased as the pump power was increased. When the power went up to 200 mW, the tumor temperature reached above 43 °C, which was a proper temperature to necrotize the tumor cells while maintaining the normal tissues within minimal damage. Then, we had recorded an in vivo PTT cycle in real time (Figure 5d). As the 200 mW pump laser was transferred to the fiber‐tip probe, and the tumor lesion area showed a rapid and clear temperature response as the pump was “off‐on,” After 250 s of treatment start, the tumor temperature remained steady at 43.5‐45 °C, and when the pump was turned off tumor temperature decreased to normal within 200 s. Moreover, during the 15 min treatment as illustrated in video S2 (Supporting Information), the photothermal imager data and the fiber‐optic temperature sensor measurements showed unanimous fluctuations to confirm the reliability of the multifunctional fiber‐based photothermal treatment strategy.

**Figure 6:**
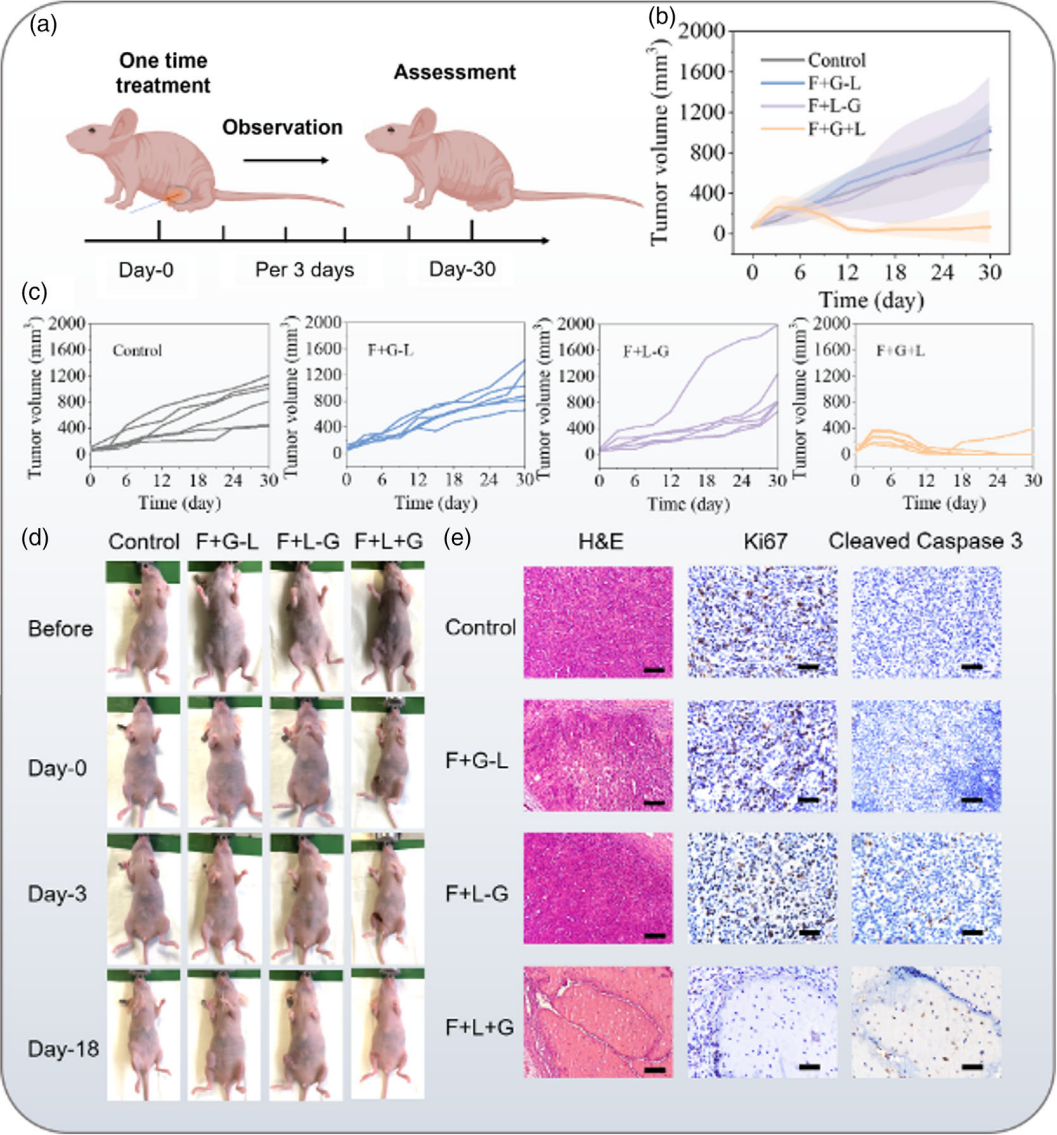
Anticancer efficacy of the fiber PTT. a) Scheme of one‐time PTT in vivo strategy. b,c) Tumor growth inhibition curves (the volume (mm^3^) = 1/2 × (tumor length) × (tumor width)^2^ of different groups (control, “F + G‐L”, “F + L‐G,” and “F + G + L” group) of tumor‐bearing mice after treatments (*n* = 6). d) Representative photographs of mice bearing MDA‐MB‐231 xenografts in different group sets before and after various treatments. Control group: control without any treatment; “F + G‐L” group: Gr/AuNS optical fiber without laser injection; “F + L‐G” group: the bare optical fiber with 200 mW laser injection; “F + G + L” group: the Gr/AuNS optical fiber with 200 mW laser injection. e) Histology analysis of therapeutic effects in MDA‐MB‐231 tumor sections regarding the different treatments and periods (*n* = 6). The scale bars of H&E are 100 μm, and the scale bars of Ki67 and Cleaved‐caspase 3 are 50 μm.

Then, the therapeutic efficacy of the iFOT probe was further evaluated by the in vivo group study of tumor‐bearing mice. The female BALB/c nude mice bearing MDA‐MB‐231 tumors (20 g of average body weight and 70 mm^3^ of average tumor volume) were randomly assigned to four groups, including control, Gr/AuNS fiber without laser radiation(F + G–L), bare fiber with laser radiation (F–G + L), and Gr/AuNS fiber with laser radiation (F + G + L). The “F + G–L” and “F–G + L” groups were set to rule out the independent variable influence of the graphene and laser. We conducted only one operation of treatment lasting for 15 min for each mouse. The tumor volumes and body weights of the mice were observed continuously every 3 days (**Figure** **6**a). Figure 6b,c shows the evolution of tumor volume after treatment in each group, respectively. The mice in the “F + G + L” group displayed significant tumor suppression due to the PTT effect. The average tumor volume was approximately only 7.62% of the blank group at the 30 days after treatment. Quantitative analysis reveals the final efficacy of PTT at day 30 (Figure S17, Supporting Information). However, intriguingly, the tumor volume of partial controlled groups grew faster probably due to the stress reaction of the tumor after the exogenous stimuli. It implies a considerable antitumor effect of PTT using Gr/AuNS fiber probe, as is further demonstrated in continued individual recordings of mice (Figure 6d). The mice of “F + G + L” group exhibited visible ablative marks on the flank immediately after treatment and formed eschars after 2 days. After 18 days, the eschars fell off and no signs of tumor recurrence were observed in the mice of the treatment group, with a high complete cure rate of 83.33%. By contrast, the mice in the controlled groups did not show any eschar and tumors were not suppressed after treatment. Therefore, fiber puncture, Gr/AuNS materials, and laser cannot eradicate solid tumors independently.

To verify the therapeutic effects of the PTT strategy, further histological reports were developed as shown in Figure 6e. Hematoxylin and eosin (H&E) staining showed that the most severe tumor cell damages were observed in the group of “F + G + L,” while control groups had much lower degree of damage. Moreover, in order to further reveal the mechanism of PTT, the Ki67^+^ tumor cells and Cleaved‐Caspase 3^+^ tumor cells in each group were examined by immunohistochemical staining. Compared to other groups, the number of proliferative tumor cells was significantly decreased while the apoptotic tumor cells were drastically increased in the group of “F + G + L.” These results indicate that fiber PTT can effectively kill tumor cells and suppress tumor proliferation to achieve the purpose of anticancer treatment.

**Figure 7:**
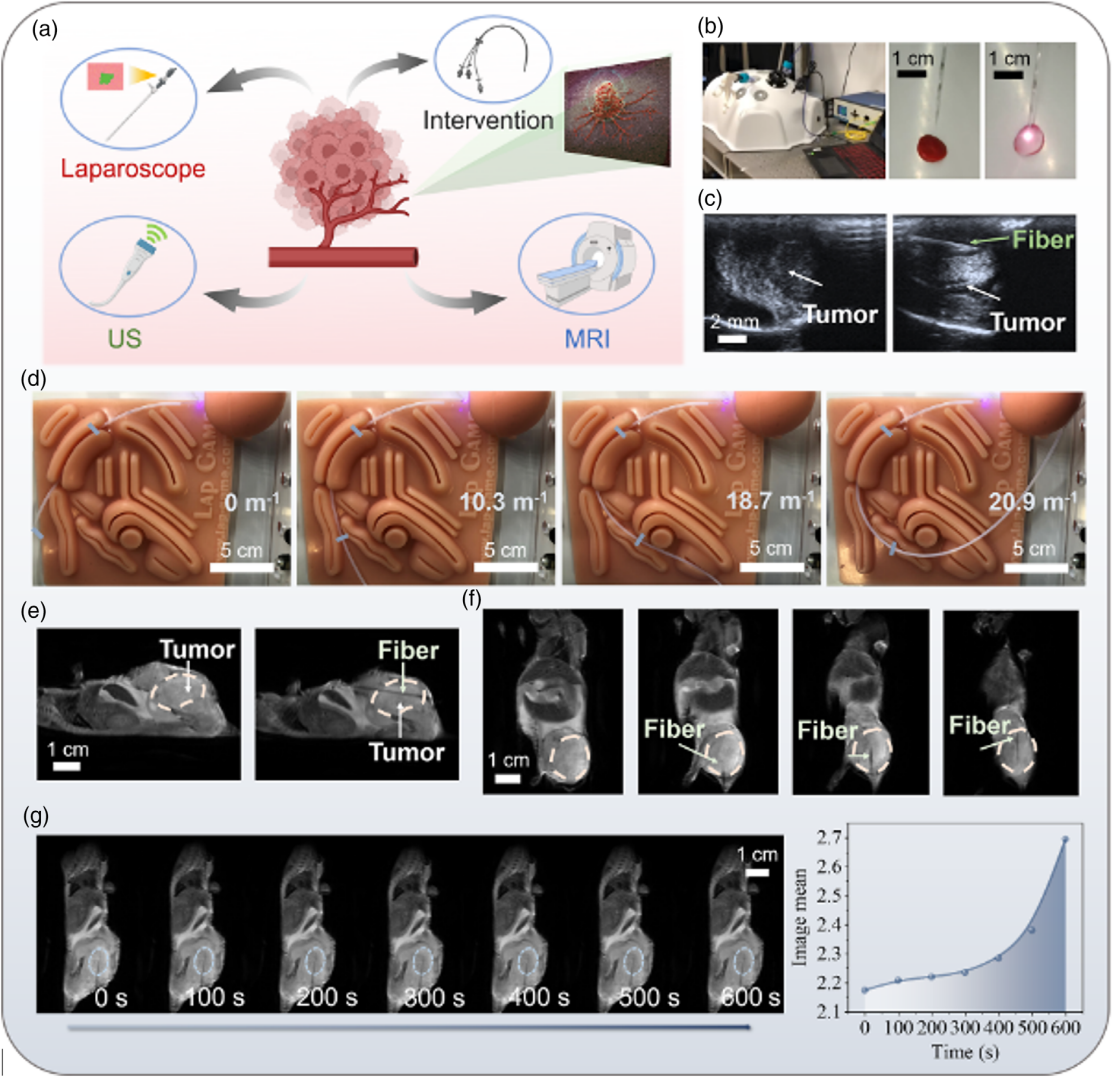
Optical fiber and image combination platform for biological applications. a) Scheme of the compatibility of iFOT with current imaging technology. b) Endoscopic guidance assisted in the laparoscopic simulation of tumor treatment experiment. The red temperature‐sensitive material sensing the high temperature of Gr/AuNS fibers caused the surface color variation, which can be observed clearly in the endoscope lens. c) Positioning of the fiber probe based on US guidance in the tumor of mice. d) The interventional theranostics of iFOT using phantom vessel with different curvatures. e) Positioning of the fiber probe based on MRI guidance in the tumor of mice. f) The movement of optical fibers in mouse tumors with MRI monitoring for 1 min. g) Continuous MRI monitoring of tumor tissue in 10 min (recorded per 100 s) to confirm the reliability of the optical fiber PTT strategy. And evolution of MRI signals in the solid tumor.

To cope with the deep‐seated lesion of the tumor, endoscope and intervention operation are feasible pathways for driving the fiber‐optic theranostic probe into practice. However, the guidance of optical fiber to the appropriate position of tumor remains challenging. Fortunately, regarding the anti‐interference nature and pliability of optical fiber, the medical imaging technologies can greatly facilitate the navigation of the optical fiber in this new strategy. For example, the US‐guided optical needles probe,^[^ ^23^ ^]^ the PAI‐guided percutaneous needles,^[^ ^24^ ^]^ and the optically controlled MR‐compatible active needle^[^ ^25^ ^]^ are excellent paradigms that orchestrate the optical fiber and image navigation technologies. Therefore, in order to assess the viability of merging the proposed iFOT with the medical imaging techniques, several routinely used imaging methods were tested, including endoscopy, US, PAI, and MRI in **Figure** **7**a. As shown in Figure 7b, at first, the optical fiber could be flexibly manipulated using the endoscopic trocar that was integrated by a medical laparoscopic training equipment. The thermochromic material was deployed inside the abdominal cavity phantom to reveal the photothermal effect of Gr/AuNS fiber. Furthermore, as shown in video S3 (Supporting Information), the color variation according to moving track of fiber probe heater (following the handwriting as “J‐N‐U”) on the thermochromic plate was confirmed by the temperature value indicated by the distributed FBG sensors. The visibility and controllability revealed the ability of the iFOT probe to perform as an endoscopic diagnostic and therapeutic tool in minimally invasive surgery. In the following, the iFOT probe, which was intervened into the tumor of mouse in vivo, was displayed under US imaging, as shown in Figure 7c. The tumor morphology and location of the intervened fiber could be clearly observed in the field of view. A video of fiber that slowly withdrew from the tumor was recorded to demonstrate the real‐time feedback of the position of the iFOT regarding the tumor under the US imaging (Video S4, Supporting Information). In addition, the iFOT could as well gain the assistance under PA imaging (Figure S18, Supporting Information).

The medical care compatibility of iFOT was also demonstrated by employing the theranostic probe in the minimal‐invasive interventional surgery for deep lesions. As shown in a phantom test (video S5, Supporting Information), iFOT can be easily conducted into the microcatheter to navigate the natural channel inside the body, such as blood vessel of artery and vein, for targeting tumor model. After passing the curved soft tube mimicking blood vessel through the microcatheter, the probe reached the tumor phantom, and the optical diagnosis and photothermal treatment could be subsequently performed. Thanks to the flexibility and robustness, iFOT can be crossed through microcatheters and vascular models with different curvatures from 0 to 20 m^−1^, as shown in Figure 7d. The results reveal that the iFOT can unleash its potential in interventional treatment for tumor, which would have been out‐of‐reach.

Finally, we demonstrated further possibilities for in situ diagnosis and treatment of the iFOT under MRI taking advantage of the electric isolation and anti‐EM interference characteristics of optical fiber. Similar to the above imaging techniques, MRI could also visualize the tumor and punctured fiber tip in vivo (Figure 7e). Meanwhile, the MRI supports 360 degree scanned images and continuous monitoring with a stable field of view. As shown in Figure 7f and video S6 (Supporting Information), the entire process of fiber movement in the tumor was recorded by MRI. The high‐resolution images provided by MRI enable accurate targeting and positioning of the fiber, ensuring that iFOT can effectively focus on the lesion. Furthermore, the optical fiber pathway enables the possibility of remote sensing and controlling, which can bridge the magnetic resonance receiving coil and optoelectronic instruments, the latter of which are vulnerable under the extremely strong magnetic field (Figure S19, Supporting Information). While performing fiber PTT on the tumor of mice, we used MRI to monitor the tumor changes in mice in real time (Figure 7g). The continuous scan with a total scan time of 10 min (assessed every 100 s) was conducted. To perform a quantitative analysis of tumor T2‐weighted signal intensity (SI), we delineated region‐of‐interests (ROIs) at the center of tumor. The average SI of these ROIs was computed and subsequently normalized using the SI of the adjacent normal tissues. As the PTT continued, the tumor in the vicinity of the fiber tip turned darker in the image and the SI decreased from 2.69 to 2.17 in Figure 7g, which indicated the decrease of hydrogen ions that was caused by heat‐mediated tumor dehydration. With the support of the image navigation terminal, we were able to guide the fiber probe to the deeper tumor lesions in vivo. Image‐assisted fiber probes can accurately detect the density of tumor cells and even identify tumor margins. The dual tumor diagnosis strategy further enhances the success rate of PTT. Hence, regarding the high compatibility of the iFOT with the medical imaging techniques, the combination of iFOT probes and imaging tools can be an important advance in ensuring the efficiency and biosafety of the action of the optical fiber probe in vivo.

## 3 Discussion

In summary, we demonstrated the integrated strategy for the diagnosis and treatment of tumors. The optical fiber was developed as a probe with integrated theranostics functions, enabling tumor sensing and PTT just by fluorescent molecules and Gr/AuNS nanomaterials. iFOT probe not only can distinguish the cancerous site from the normal tissue within 10 s, but also has the ability to identify the marginal tissue surrounding tumors. Meanwhile, the PPT with low‐power pump laser eliminates the mouse tumor at one time by the same iFOT probe. This strategy provides a “plug‐and‐play” paradigm of operation, and can play an important role to upgrade the traditional optical technologies for cancer theranostics. In addition, the compact iFOT probe has high compatibility with minimally invasive interventional surgery and MIR & US technologies in clinical medicine, allowing in vivo guidance to deeper lesions for tumor diagnosis and treatment. It denotes that iFOT probe can participate in the whole process of cancer theranostics including early diagnosis, intraoperative visualization, immediate treatment, and postoperative monitoring, thus improving the cure rate and reducing the burden on patients. In the future research, it is essential to design multimodal (fluorescence, Raman, autofluorescence, refractive index) and multiparametric (temperature, pH, hypoxia, tumor‐specific proteins) cancer sensing, and develop combination therapies (PTT, PDT, immunotherapy, gene therapy) to tackle the tricky tumors with high atypia and heterogeneity. With remarkable biosafety, multiexpansion, and high compatibility of medical technology, the iFOT probe is promising to be broadly applied in the field of medical oncology.

## 4 Experimental Section

4.1

4.1.1

##### Surface Pretreatment and Synthesis of the Nanocomposites

The graphene (Nanjing XF NANO Materials Tech Co. Ltd., China) was typically 8–15 nm in sheet diameter and 10–50 μm in thickness. Prior to the AuNS deposition, the graphene was pretreated for activation procedures. The graphene was treated with acid to introduce active functional groups onto the inert graphene surface. 0.05 g of pristine graphene was added into a mixture of 15 mL concentrated HNO_3_ (Guangzhou Rongman Biotechnology Co., Ltd., China, 68%, AR) and 5 mL concentrated HSO_4_ (Guangzhou Rongman Biotechnology Co., Ltd., China, 98%, AR) for 1 h at room temperature. The graphene suspension in acid mixture was heated to 80 °C and kept stirring for 5 h using oil bath. Then, the graphene suspension was filtered and washed thoroughly with deionized water until it was neutral. The surface‐activated graphene was dried in an oven overnight at 60 °C before use.

AuNS were prepared as described in previous work using a seed‐mediated method.^17^ Typically, following the addition of 100 μL of the gold seed (Najing Technology Co., Ltd., China, 0.1 nM), 50 μL of 0.1 M ascorbic acid (Shanghai Aladdin Biochemical Technology Co., Ltd., China, 99.0%, AR) and 50 μL of AgNO_3_ (Shanghai Macklin Biochemical Co., Ltd., China, 0.1 N) were added simultaneously while stirring moderately to a 10 mL solution of 0.25 mM HAuCl_4_ (Shanghai Macklin Biochemical Co., Ltd., China, 50% Au basis) containing 10 mL of 1 N HCl (Guangzhou Rongman Biotechnology Co., Ltd., China, 36%, AR). The concentration of AgNO_3_ controls the branch number and branch length of AuNS. Finally, the Gr/AuNS mixture was completed to redispersed surface‐activated graphene in the above solution.

##### The Interaction Between Gr and AuNS

After the pretreatment of Gr and AuNS, the Gr surface is enriched with functional groups (–COOH, –OH, and –C–O–C–), while the AuNS surface is also linked with –OH groups. Due to the presence of oxygen‐containing functional groups such as –COOH and –OH, graphene carries a negative charge, while the hydroxyl groups on the AuNS surface can be protonated in an acidic environment and form a positive charge. Therefore, the two materials combine through electrostatic adsorption.

However, there is the aggregation of AuNS, which occurs due to the high surface area of AuNS, resulting in very high surface energy. To reduce surface energy, the AuNS tend to aggregate, reducing the free surface and thus the total energy of the system. However, the physical adsorption forces between Gr and AuNS are relatively weak, and they cannot effectively prevent the mutual attraction and aggregation between AuNS, resulting in a low binding density on Gr. Fortunately, these do not affect the functionalization of the optical fiber by Gr and AuNS.

##### Characterization of Materials

The morphology of the samples was measured by field‐emission SEM (Apreo 2 SEM) and TEM (TF20). The structure of the samples was characterized by powder XRD (D8, ADVANCE) with Cu Kα radiation and Raman spectrometer (LabRAM HR Evolution) with an excitation wavelength of 532 nm. UV–vis absorbance spectra of samples were observed by UV–vis–NIR spectrophotometer (SHIMADZU, UV‐3600 plus, Japan).

##### Cell Lines and Cell Culture

Human triple‐negative breast cancer cell line MDA‐MB‐231 was obtained from the American Type Culture Collection and cultured at 37 °C in a humidified atmosphere containing 5% CO_2_ in Dulbecco's modified Eagle medium (Gibco, Grand Island) with 10% fetal bovine serum (ExCell Bio, Shanghai, China) and 1% penicillin–streptomycin (Gibco). The cells utilized in this investigation were verified to be free of cross‐contamination from other human cell lines using the Short Tandem Repeat (STR) Multi‐Amplification Kit (Microreader 21 ID System) and negative for mycoplasma using the Mycoplasma Detection Set (M&C Gene Technology, Beijing, China).

##### Animals

The 4–6 week old BALB/c‐Nu mice [BALB/cJGpt‐Foxn1nu/Gpt] were purchased from GemPharmatech (Nanjing, China). Animal experiments were approved by Jinan University's Institute of Experimental Animal Ethics Committee (Approval number: IACUC‐20220923‐01), and all mice were kept in a pathogen‐free environment.

##### Establishment of Orthotopic Breast Cancer Xenografts

Human triple‐negative breast cancer cell line MDA‐MB‐231 (5 × 10^5^ cells) suspended in PBS (200 μL) was inoculated into the fourth mammary fat pad of BALB/c‐Nu mice (7 weeks old, female). When the tumor size reached 70 mm^3^, tumor‐bearing mice were randomly divided into four groups (*n* = 8): 1) the Gr/AuNS fiber with 200 mW laser injection (“F + G + L” group), 2) the Gr/AuNS fiber without laser injection (“F + G–L” group), 3) the bare optical fiber with 200 mW laser injection (“F + L–G” group), and (d) control without any treatment (control group). After 12 h posttreatment, two tumors in each group were randomly selected, weighed, photographed and stained with H&E. Body weight and tumor volume of other mice were measured and recorded every 3 days. The tumor volume was calculated according to the following equation: *V* = *a* × *b* ^2^/2 (*a* was the tumor length and *b* was the tumor width).

##### Histological and Immunohistochemical Analyses

The paraffin‐embedded tumor tissues were sectioned at a thickness of 5 μm. Histological and apoptotic cells in tumor tissues were evaluated using H&E staining. For immunohistochemical analysis, sections were deparaffinized. And antigen retrieval was performed using ethylenediaminetetraacetic acid antigen retrieval solution (Beyotime). The slides were blocked with 5% bovine serum albumin for 1 h. Subsequently, they were mixed with anti‐CD31 (cat. AF3628, 1:200 dilution, R&D Systems), anti‐Ki67 (cat. 9449, 1:200 dilution, Cell Signaling Technology), and anti‐Cleaved caspase 3 (cat. 9664, 1:200 dilution, Cell Signaling Technology) antibodies overnight at 4 °C. The slides were washed with PBS and incubated with horseradish peroxidase (HRP)‐coupled secondary antibodies, including HRP‐coupled anti‐mouse (Cat. 7076, 1:400, Cell Signaling Technology), antirabbit (Cat. 7074, 1:400, Cell Signaling) technology, and goat (cat. HAF019, 1:400 dilution, R&D Systems). Then we stained with a diaminobenzidine kit followed by hematoxylin restaining. The images were captured with an Olympus BX 53 microscope and analyzed with Image‐Pro Plus 6.0 software.

##### Design and Preparation of the Fiber for Diagnosis and Treatment of Tumor

The integrated solution for direct cancer sensing and therapy are detailed in our previous articles.^[^ ^5^, ^8^ ^]^ Here, we employed a different approach to enable the deployment of flexible, scalable, and easy‐to‐use optical fiber with tumor diagnostic and thermal therapy capabilities. Hydrofluoric acid (Guangzhou Chemical Reagent Factory, China, 40%, AR) showed different corrosion rates on bare and wrapped fibers due to the protection of the fiber‐optic polymer coating. After the duration of 2.5 h etching, the fiber would present a cone‐column structure (150–400 μm). Then the remaining coating layer was removed and the fiber end was tailored as a cone. To functionalize the fiber, steps including hydroxylation, salinization, and Gr/AuNS immobilization were indispensable. The Gr/AuNS was anchored to the fiber by electrostatic adsorption and hydrogen bonding. Finally, iFOT probe was completed by chemical bonding of the NTR probe onto the fiber.

##### In Vitro Fluorescence Sensing Test

Gr/AuNS suspensions with different AuNS concentrations (0, 0.025, 0.05, 0.075 and 0.1 nM) were prepared separately at constant Gr content, respectively. To obtain the modified fiber tip with different sensitivities, Gr/AuNS and fluorescent molecules were immobilized on the fiber tip. The fluorescence of fluorescent molecules was stimulated by the 450 nm excitation laser (customized by the Shenzhen Innova Optoelectron. Tech., China, 450 nm laser diode) through the functional fiber tip, and the fluorescence signal was collected by the spectrometer (purchased from Ocean optic, QE pro, USA). Vitally, a 450 nm filter was set before the receiver to exclude excessive excitation light intensity. For determining the appropriate AuNS content in the Gr/AuNS solution, we tested the sensitivity with the same PBS‐NTR‐fluorescent molecules mixture and in the same light source.

In addition, the tumor fiber sensor was calibrated. Pure PBS buffer solution and the mixtures with different concentrations of NTR were prepared (0, 5, 10, 20, 30, 50, 100, 200, 500, and 1000 ng mL^−1^), respectively. The spectrum was recorded 3 times in the PBS‐NTR‐fluorescent molecules mixtures at 37 °C to portray error lines. Since the filter filtered almost all the excitation light, the spectrum showed a fluorescent signal near 550 nm accompanied by a small amount of faint excitation light signal. Thereafter, the PBS‐NTR‐fluorescent molecules mixtures were utilized as the samples, and the fluorescence spectra corresponding to different NTR concentrations were obtained by subtracting the pure PBS spectrum (baseline). After normalizing the values of the spectrum, the values were fitted by the calibrating functions. The LOD and LOQ were determined according to the following equation:

(1) $$X_{\text{LOD}}=f^{-1}\left(\bar{y_{\text{blank}}}+10/3\times \sigma \right)$$

(2) $$X_{\text{LOQ}}=f^{-1}\left(\bar{y_{\text{blank}}}+10\times \sigma \right)$$

where (f) is the calibrating function and ($\bar{y_{\text{blank}}}$ ± σ) is the blank sample data.

##### In Vivo Detection of Tumors

Tumor fluorescence signals from mice were detected using the fiber tip immobilized with Gr/AuNS and fluorescent molecules. Due to the needle cone structure of the fiber tip, the fiber can directly puncture the skin of mice. To discriminate, the same part of the lateral abdomen of the normal mouse (normal tissue) and the tumor‐bearing mouse (tumor tissue) were detected separately. In each group, the fluorescence signal was detected by spectroscopy for 1 min with the same method. A group of data was recorded every 10 s (M > 3). After the normalization of the spectral lines, the fluorescence signals could be clearly distinguished by the spectrum. Statistical analysis was performed between the tumor data recorded at 0 and 60 s and normal tissue data at 60 s. The experiment was repeated using the fibers with the same preparation process, and the fiber tip was enabled circularly by piranha solution washing and secondary functionalization.

##### In Vitro Photothermal Property Evaluation

To prepare different concentrations of Gr/AuNS suspensions separately, the graphene was added into the Gr/AuNS suspension with concentrations of 0, 0.1, 1, 2, 5, and 10 mg mL^−1^ graphene. Subsequently, the fiber heads were functionalized using Gr/AuNS suspensions with different graphene concentrations. Since the 980‐pump laser light (Shenzhen Innova Optoelectron. Tech., China) was transported to the therapy fiber, the Gr/AuNS on the fiber tip surface generated heat in the presence of pump laser. And the Gr/AuNS on the fiber tip surface showed different photothermal conversion efficiency. IR imaging during the photothermal test was acquired by the photothermal imager (FOTRIC 225s, FOTRIC, China) to reference the fiber functionalization modality.

##### In Vivo PTT

Before formal treatment, photothermal testing was performed on live tumor‐bearing mice to obtain thermal actuation data. Isoflurane (RWD Life Science Co. Ltd., China) was guided into tumor‐bearing mice using a small animal anesthesia machine (R580S, RWD Life Science Co. Ltd., China). The flow rate of isoflurane was 2 L min^−1^ and the density was 1.5%. To enable the thermal range of the fiber tip can cover the entire tumor, the proper angle and penetration depth were selected necessarily. The 980 nm laser with power of 0, 50, 100, 150, 200, and 250 mW were transported through the fiber and the tumor was damaged using the photothermal effect of Gr/AuNS. Real‐time thermal dates of the tumor were obtained simultaneously using the photothermal imager and optical fiber temperature sensor, and quantified by BM_IR and MATLAB software. After confirming the treatment strategy, the fiber tip was inserted into the tumor of the mouse for a 15 min treatment procedure. Disinfection was carried out before and after each operation. Further, PTT was further evaluated by recording tumor volumes and body weights.

##### US Scan Parameters

The TOSHIBA Aplio500 US diagnostic instrument was used; the probe frequency was selected to be 18 MHz and the imaging depth was adjusted to 2 cm. The mice were fixed on the examination bed and the coupling agent was applied. The US probe was parallel to the mice's body, and the tumor was scanned in longitudinal and transverse sections to clarify the tumor boundary and measure the tumor size. Subsequently, the position of the fiber probe was shown and the process of fiber probe entry into the tumor was dynamically observed.

##### PAI Scan Parameters

The 532 nm/558 nm dual‐wavelength light source was used for photoacoustic excitation. The 558 nm laser beam was generated by the single‐mode fiber (HB450‐SC, Fibercore) with the 532 nm nanosecond laser (VPFL‐G‐20, Spectra‐Physics, Inc.) via the stimulated Raman scattering effect.^[^ ^24^ ^]^ The dual‐color pulse trains were interleaved with a time delay of 2000 ns for mouse tumor imaging to minimize the effect of the artifact. The frequency was selected to be 10 MHz; the imaging depth and length were adjusted to 50 mm and 25 μm, separately.

##### MR Scan Sequences and Parameters

Scanning was performed with the GE750 3.0TMR machine with small animal coils. In vivo imaging was obtained on the tumor‐bearing mouse model, and the mice were placed in the coil in a supine position after anesthesia. The mouse body was parallel to the BO field while the center of its tumor was placed in the center of the coil and the magnet. 1) Fast spin‐echo T1WI: axial scanning, TR = 625.0 ms, TE = 9.4 ms, layer thickness=1.6 mm, layer spacing = 0.1 mm, FOV = 10 × 10 cm, matrix = 288 × 288, NEX = 2, bandwidth = 31.25 kHz, ETL = 3. 2) Fast spin‐echo T2WI: axial scanning, TR = 3000.0 ms, TE = 68.0 ms, slayer thickness=1.6 mm, layer spacing = 0.1 mm, FOV = 10 × 10 cm, matrix=288 × 288, NEX = 4, bandwidth = 15.63 kHz, ETL = 14. 3) BOLD imaging: Multi‐Echo FSPGR sequence, axial scan, TR = 500 ms, TE = 38.1 ms, layer thickness = 2.2 mm, layer spacing = 0.2 mm, FOV = 8 × 8 cm, matrix=160 × 160, flip angle = 20°, NEX = 2, bandwidth = 31.25 kHz, localization line consistent with T1WI and T2WI.

##### Image Reprocessing

The GE ADW4.5 image postprocessing work station was used to obtain a pseudocolor image of the R2* value, which reflects the quantitative index of tissue oxygen content, by analyzing the raw BOLD images. Referring to the tumor T1WI and T2WI images, the region of focal was outlined on the R2* pseudocolor map to obtain quantitative indicators of oxygen content in different regions of the tumor, and three measurements were averaged.

##### Simulation of Fiber Thermal Actuation

The heating area mediated by the Gr/AuNS fiber was simulated using COMSOL. As the 980‐pump laser was input to the PTT probe, the laser impinged on the Gr/AuNS at the fiber surface to excite its photothermal effect by Gr/AuNS nanomaterials. The major pump light energy was converted into heat at Gr/AuNS nanomaterials of the fiber surface. The thermal energy then rapidly diffused from the outer diameter of the fiber, which thermalized the surrounding tumor tissue through heat conduction and a small amount of body fluid convection. The PTT of optical fiber was realized by creating necrotic areas in the tumor.

The heat source model inside the PTT fiber probe can be represented as follows:^[^ ^26^ ^]^

(3) q(r)=η·α·P(r,z)

where *⋅* is the thermal conversion efficiency, *r* is the radii of the optical fiber, respectively, *α* is the pump‐light absorption coefficient, and P(r,z) is the spatial distribution of the pump light in the fiber, which can be expressed as follows:

(4) $$P\left(r,z\right)=P_{0}\cdot P_{t}\cdot \exp\left(-\alpha \cdot z\right)$$

where $P_{0}$ is the incident pumping power and $P_{t}$(*r*) is the normalized energy distribution of the core fundamental mode.

Due to the fact that the actual thermal conductivity range is much larger than the core area acting as a heat source, the model is simplified in the simulation. The optical fiber probe was saturated with pump absorption at a diameter of 150 μm, corresponding to the maximum thermal energy generated in the range. Here, the model can be considered a simple 2D axisymmetric model and the core region generates maximal heat in the radial direction. When the input power *P*_0_ was set at 200 mW, calibrated by the power meter, through a 0.5 cm Gr/AuNS fiber, over 160 mW was converted into thermal energy. As shown in Figure S20 (Supporting Information), the temperature distribution of the photothermal probe penetrating the human tumor along the radial direction was simulated using a finite element analysis model. The other relevant parameters are as follows: *k*_glass_ = 1.4 W m^−1^ K^−1^, *C*_pglass_ = 730 J kg^−1^ K^−1^, *ρ*_glass_ = 2210 kg m^−3^; *k*_graphene/AuNS_ = 350 W m^−1^ K^−1^, *C*_pgraphene/AuNS_  = 1400 J kg^−1^ K^−1^, *ρ*_graphene_ = 2220 kg m^−3^; *k*_tumor_ = 0.52 W m^−1^ K^−1^, *C*_ptumor_ = 3540 J kg^−1^ K^−1^, *ρ*_tumor_ = 1079 kg m^−3^, *ρ* is the density of the material, *C*_p_ is the specific heat capacity at atmospheric pressure, and *k* is the thermal conductivity. The model was set with a radius of 2 cm as the boundary of the tumor tissue and the probe heating area was located at the center. The initial temperature of all positions was set at 308.15 K (35 °C).

##### Statistical Analysis

All experiments were independently repeated at least 3 times, and all data were shown as means ± standard error of the mean. Statistical analysis was performed using one‐way ANOVA followed by Tukey's post hoc test by GraphPad Prism 7.0 (GraphPad Software, USA). *P* < 0.05 was considered statistically significant.

## Conflict of Interest

The authors declare no conflict of interest.

## Author Contributions

**Bai‐Ou Guan**, **Xiangran Cai**, **Minfeng Chen**, and **Yang Ran** conceived the project. **Yang Ran**, **Fangzhou Jin**, and **Minfeng Chen** designed the experiments. **Fangzhou Jin** and **Zhiyuan Xu** performed the experiments. **Minfeng Chen** and **Dongmei Zhang** performed the modeling of xenografted animals and histology analysis. **Wei Wang** designed the nitroreductase fluorescent probes. **Zesen Li** synthesized the nitroreductase fluorescent probe and related samples. **Yang Wu** established the fluorescent sensing platform. **Fangzhou Jin**, **Xu Yue**, and **YongKang Zhang** performed SEM, TEM, XRD, Raman, and UV–vis characterizations. **Zhongyuan Cheng** and **Youzhen Feng** provided MRI images. **Wei Li** provided PAI images. **Yang Ran**, **Fangzhou Jin**, and **Minfeng Chen** validated the results. **Fangzhou Jin** and **Zhiyuan Xu** performed the numerical analysis of the fiber thermalization. **Yang Ran**, **Fangzhou Jin**, **Xiangran Cai**, **Minfeng Chen**, and **Bai‐Ou Guan** wrote the manuscript. **Yang Ran**, **Minfeng Chen**, **Bai‐Ou Guan**, and **Donglin Cao** supervised the investigation. All authors analyzed the data, discussed the results, and commented on the paper.

## Supporting information

Supplementary Material

## Data Availability

The data that support the findings of this study are available from the corresponding author upon reasonable request.

## References

[1] a) H. Jin , L. Wang , R. Bernards , Nat. Rev. Drug Discovery 2023, 22, 213; 36509911 10.1038/s41573-022-00615-z

[2] a) Z. Xie , T. Fan , J. An , W. Choi , Y. Duo , Y. Ge , B. Zhang , G. Nie , N. Xie , T. Zheng , Y. Chen , H. Zhang , J. S. Kim , Chem. Soc. Rev. 2020, 49, 8065; 32567633 10.1039/d0cs00215a

[3] a) J. E. Fröch , L. Huang , Q. A. Tanguy , S. Colburn , A. Zhan , A. Ravagli , E. J. Seibel , K. F. Böhringer , A. Majumdar , eLight 2023, 3, 1;36618904

[4] a) A. L. Chin , S. Jiang , E. Jang , L. Niu , L. Li , X. Jia , R. Tong , Nat. Commun. 2021, 12, 5138; 34446702 10.1038/s41467-021-25391-z PMC8390758

[5] Y. Wu , M. Chen , J. Cai , Z. Xu , F. Jin , Y. Zhang , W. Wang , Y. Ran , D. Zhang , B.-O. Guan , IEEE Sens. J. 2022, 22, 22646.

[6] G. Chen , K. Hou , N. Yu , P. Wei , T. Chen , C. Zhang , S. Wang , H. Liu , R. Cao , L. Zhu , Nat. Commun. 2022, 13, 7789. 36526631 10.1038/s41467-022-35440-w PMC9758120

[7] K. Deng , Y. Tang , Y. Xiao , D. Zhong , H. Zhang , W. Fang , L. Shen , Z. Wang , J. Pan , Y. Lu , Nat. Commun. 2023, 14, 3069. 37244895 10.1038/s41467-023-38554-x PMC10224912

[8] Y. Ran , Z. Xu , M. Chen , W. Wang , Y. Wu , J. Cai , J. Long , Z. S. Chen , D. Zhang , B. O. Guan , Adv. Sci. 2022, 9, 2200456. 10.1002/advs.202200456 PMC9130922 35319824

[9] H. Wu , P. Chen , X. Zhan , K. Lin , T. Hu , A. Xiao , J. Liang , Y. Huang , Y. Huang , B. O. Guan , Adv. Mater. 2023, 36, 2310571. 10.1002/adma.202310571 38029784

[10] a) S. Song , Y. Zhao , M. Kang , F. Zhang , Q. Wu , N. Niu , H. Yang , H. Wen , S. Fu , X. Li , Adv. Mater. 2024, 36, 2309748; 10.1002/adma.202309748 38165653

[11] a) Z. Zhan , M. Cantono , V. Kamalov , A. Mecozzi , R. Müller , S. Yin , J. C. Castellanos , Science 2021, 371, 931; 33632843 10.1126/science.abe6648

[12] a) A. Canales , X. Jia , U. P. Froriep , R. A. Koppes , C. M. Tringides , J. Selvidge , C. Lu , C. Hou , L. Wei , Y. Fink , P. Anikeeva , Nat. Biotechnol. 2015, 33, 277; 25599177 10.1038/nbt.3093

[13] F. Giurazza , F. Corvino , M. Silvestre , G. Cangiano , G. De Magistris , E. Cavaglià , F. Amodio , R. Niola , La Radiol. Med. 2021, 126, 474. 10.1007/s11547-020-01274-z 32889705

[14] a) K. Yang , L. Feng , X. Shi , Z. Liu , Chem. Soc. Rev. 2013, 42, 530; 23059655 10.1039/c2cs35342c

[15] a) H. Yuan , H. Zhang , K. Huang , Y. Cheng , K. Wang , S. Cheng , W. Li , J. Jiang , J. Li , C. Tu , ACS Nano 2022, 16, 2577; 35107258 10.1021/acsnano.1c09269

[16] a) Y. Liu , H. Yuan , A. M. Fales , J. K. Register , T. Vo‐Dinh , Front. Chem. 2015, 3, 51; 26322306 10.3389/fchem.2015.00051 PMC4533003

[17] a) D. Hu , T. Liu , Q. Yang , Z. Xu , J. Long , Y. Ran , X.-M. Duan , B.-O. Guan , J. Lightwave Technol. 2022, 40, 4812;

[18] a) S. Mostufa , T. B. A. Akib , M. M. Rana , M. R. Islam , Biosensors 2022, 12, 603; 36004999 10.3390/bios12080603 PMC9405676

[19] F. Jin , M. Li , L. Xie , J. Jiang , J. Power Sources 2021, 514, 230587.

[20] C. Fernández‐López , C. Mateo‐Mateo , R. A. Alvarez‐Puebla , J. Pérez‐Juste , I. Pastoriza‐Santos , L. M. Liz‐Marzán , Langmuir 2009, 25, 13894. 19591480 10.1021/la9016454

[21] Y. Liu , J. R. Ashton , E. J. Moding , H. Yuan , J. K. Register , A. M. Fales , J. Choi , M. J. Whitley , X. Zhao , Y. Qi , Theranostics 2015, 5, 946. 26155311 10.7150/thno.11974 PMC4493533

[22] Y. Li , Y. Sun , J. Li , Q. Su , W. Yuan , Y. Dai , C. Han , Q. Wang , W. Feng , F. Li , J. Am. Chem. Soc. 2015, 137, 6407. 25923361 10.1021/jacs.5b04097

[23] V. Dremin , E. Potapova , E. Zherebtsov , K. Kandurova , V. Shupletsov , A. Alekseyev , A. Mamoshin , A. Dunaev , Sci. Rep. 2020, 10, 14200. 32848190 10.1038/s41598-020-71089-5 PMC7449966

[24] Y. Liang , W. Fu , Q. Li , X. Chen , H. Sun , L. Wang , L. Jin , W. Huang , B.-O. Guan , Nat. Commun. 2022, 13, 7604. 36494360 10.1038/s41467-022-35259-5 PMC9734171

[25] S. C. Ryu , Z. F. Quek , J.-S. Koh , P. Renaud , R. J. Black , B. Moslehi , B. L. Daniel , K.-J. Cho , M. R. Cutkosky , IEEE Trans. Robot. 2014, 31, 1. 10.1109/TRO.2014.2367351 PMC4620588 26512231

[26] a) L. Yan , C. H. Lee , J. Appl. Phys. 1994, 75, 1286;

