# Supplementary material for: Integrating Tumor Hypoxic Sensing and Photothermal Therapy Using a Miniaturized Fiber‐Optic Theranostic Probe
Source: Small Sci. 2024 Oct 31;5(2):2400450. doi: 10.1002/smsc.202400450 (PMC11934894; doi:10.1002/smsc.202400450)
Supplement: Supplementary file 1 — Supplementary Material [file SMSC-5-2400450-s001.pdf]

Supplementary Materials for

Integrating tumor hypoxic sensing and photothermal therapy using a miniaturized fiber-optic theranostic probe

Fangzhou Jin[1], Zhiyuan Xu[2], Wei Wang[1], Zhongyuan Cheng[3], Yang Wu[1], Zesen Li[1], Enlai Song[1], Xu Yue[1], YongKang Zhang[1], Wei Li[1], Youzhen Feng[3], Donglin Cao[2], Dongmei Zhang[4], Minfeng Chen[4*], Xiangran Cai[3*], Yang Ran[1,2*], Bai-Ou Guan[1*]

*Corresponding author. Email: tranyang@jnu.edu.cn (Y. R.), tguanbo@jnu.edu.cn (B.-O. G.), caixran@jnu.edu.cn (X. C.), minfengchen@jnu.edu.cn (M. C.)

This PDF file includes:

Materials and Methods

Supplementary Text

Figs. S1 to S20

Captions for Movies S1 to S5

References

Other Supplementary Materials for this manuscript include the following:

Movies S1 and S6 (.mp4)

Supplementary Text

Design of the Sensing fiber

Customization of the structural morphology of gold nanostars (AuNS) can determine their fluorescence-enhanced wavelengths. In this work, we used a seed-mediated method to synthesize three types of AuNS with different surface morphologies that were previously reported [1], including AuNS (S10), AuNS (S20), and AuNS (S30). The gold nanosphere (AuNts) and different AuNS were combined with graphene (Gr) to immobilize the surface of the optical fiber probe. The different gold nanostructures were combined with graphene to immobilize on the surface of the fiber optic probe, and the enhancements of fluorescence signal intentions were compared as shown in Fig. S7. AuNS (S10) showed the highest sensitization to the 450 nm fluorescence detection of nitroreductase (NTR), which determined the structure of the nanomaterials carried on the fiber probe.

Ex-vivo sensing

Before diagnosing the tumor in live mice, the preparation of fresh pork liver was performed as an *ex-vivo* experimental tissue substrate. The PBS buffer set was used as a baseline. The NTR-PBS mixtures prepared in the calibration section were separated according to the NTR concentration gradient, which was 0 ng mL^{-1}, 10 ng mL^{-1}, 20 ng mL^{-1}, 50 ng mL^{-1}, 100 ng mL^{-1}, 200 ng mL^{-1}, 500 ng mL^{-1} and 1000 ng mL^{-1}, respectively. As shown in Fig. S12, these mixtures were injected into pork liver tissue through a syringe. The fluorescence intensity was detected at the injection site with the integrated fiber-optic theranostics probe (iFOT) and evaluated sequentially as described in the calibration section. Similar to Fig. 3b, the recorded spectra showed that the stronger fluorescence intensity formed at the position with higher NTR density. The statistical

analysis was performed between 10 ng mL^{-1} (1000 ng mL^{-1}) and blank samples. Both the 10 ng mL^{-1} (P<0.0001) and 1000 ng mL^{-1} (P<0.0001) sample positions showed significantly positive results compared to the blank sample, which demonstrated the effective determination of NTR using the fiber-optic tip probes in tissue.

Detection for different positions of the tumor

Besides *in vivo* tumor diagnosis, iFOT can also identify tumor margins. Fiber optic sensing was used to intervene in different sites (central, eccentric, and marginal) of the same tumor in mice separately. As shown in Fig. S13, the fluorescence signals of the tumor sites evolved cumulatively with time, and the fluorescence intensity was proportional to the density of the tumor. Corresponding to Fig. 3e, the fluorescence intensity of the tumor center, tumor margin and normal tissue were detected with significant differences (P<0.0001). It means that iFOT can be used as a carcinoma margin diagnostic tool in intraoperative oncology operations.

***Ex-vivo* photothermal effect**

We compared the iFOT with the Er/Yb codoped fiber in the air from previous work[2] as shown in Fig. S15. The exothermic region of both is controlled within 5 mm of the fiber tip. With the same power (160 mW) input of 980 nm NIR light, it is observed that the thermal energy decreases with increasing length backward along the fiber tip top, and the thermal action range of iFOT was significantly larger than that of Er/Yb codoped fiber. In addition, the thermal interval range of iFOT can be customized due to the high controllability of the Gr/AuNS immobilized fiber mode, which indicated the great potential of thermal control strategy in the field of thermodynamics.

Evaluation of the biosafety from the concern of the residual nanomaterials

The biosafety of nanomaterials is a major focus of attention *in vivo* animal experiments. Therefore, we must verify the biosafety of iFOT. The probe was composed of silica, graphene and gold particles, which proved to be biologically harmless[3-5]. To exclude negative effects, we washed the probe sequentially with N-methyl pyrrolidone and deionized water to remove unstable particles and impurities. To verify the prepared iFOT, the probe was soaked with N-methyl pyrrolidone for 1 h. The residues in the solution were characterized and compared to 0.1 mg mL^{-1} Gr suspension. As shown in Fig. S16a, there were clear differences between the residual solution and the Gr solution in SEM and Raman spectra. No residues of macromolecular materials were observed, and no signal from Raman testing in the sample solution. The proven biosafety of iFOT represented its ability to be used as a theranostics tool for living organisms.

Mechanical stability of the functionalizing materials

To verify the mechanical stability of nanomaterials on the optical fiber, we repeatedly inserted and withdrew the fiber from the tissue. The SEM characterization, as well as sensing and photothermal tests, were performed on the fiber tip before and after the operation. The SEM images show that the repeated insertion and removal of the fiber within the tissue did not affect the morphology and structure of the surface materials. The test results also showed no difference in sensing efficiency and photothermal temperature (250mW) before and after the operation, with fluorescence retention and temperature retention in air being 99.14% and 98.23%, respectively. It demonstrates the mechanical stability of Gr/AuNS and NTR probes on the fiber surface.

Simulation of the action range of the photothermal treatment

The heating area mediated by the iFOT was simulated using COMSOL as shown in Fig. S20. The simulation results showed that the thermal energy accumulates with increasing heating time in the air and biological tissues. Compared to shorter heating times (5 min), the longer heating times (30 min) cause higher tissue temperatures and a larger area of thermal action. Heating levels were directly visible in the simulation graph to identify temperature differences in the regions. According to reports[6,7], irreversible tumor tissue damage occurred when the temperature reached 42 °C, and heating the tissue to a temperature of 42-46 °C for 10 min resulted in tumor cell necrosis. Thus, we set the 45 °C isotherm line as the approximate edge of the necrotic region. The simulation figure showed that with the probe heating for 5 min, the radius of the necrotic tissue area was only about 200 μm; with the probe heating for 30 min, the radius of the necrotic tissue area expanded to about 300 μm. However, the actual treatment result was better, probably because the PTT enhanced the thermal range of action by modifying the medium with the evaporation of tissue fluid.

Supplementary figures:

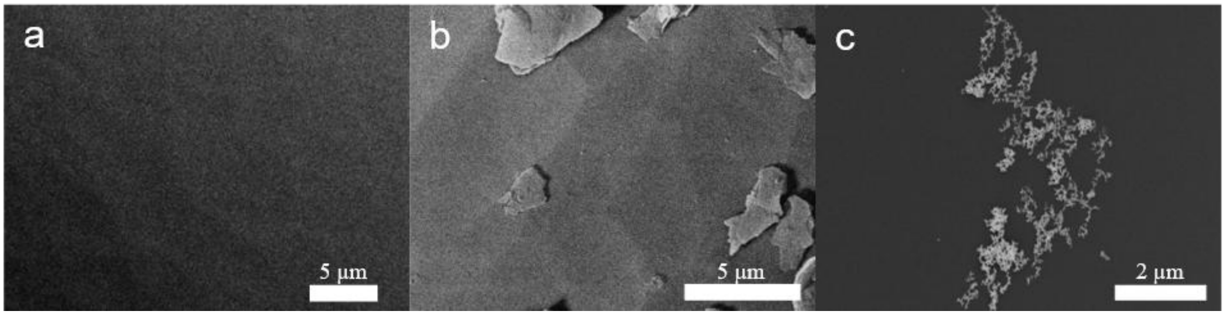

Fig. S1.

SEM images. (A) pure optical fiber. **(B)** graphene on optical fiber. **(C)** AuNS on optical fiber.

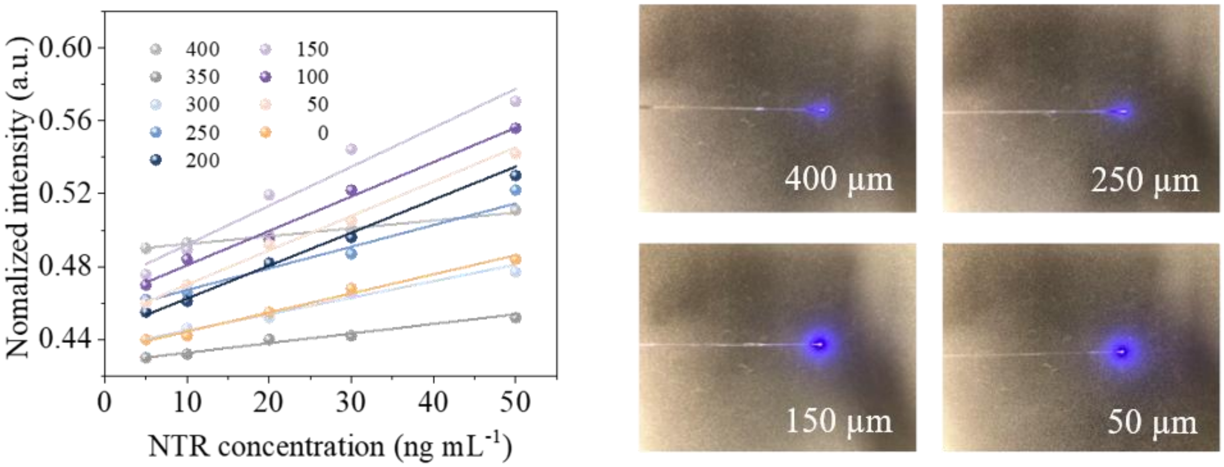

Fig. S2.

Sensing performance and calibration of the different end diameters of optical fiber tip for detecting NTR in vitro.

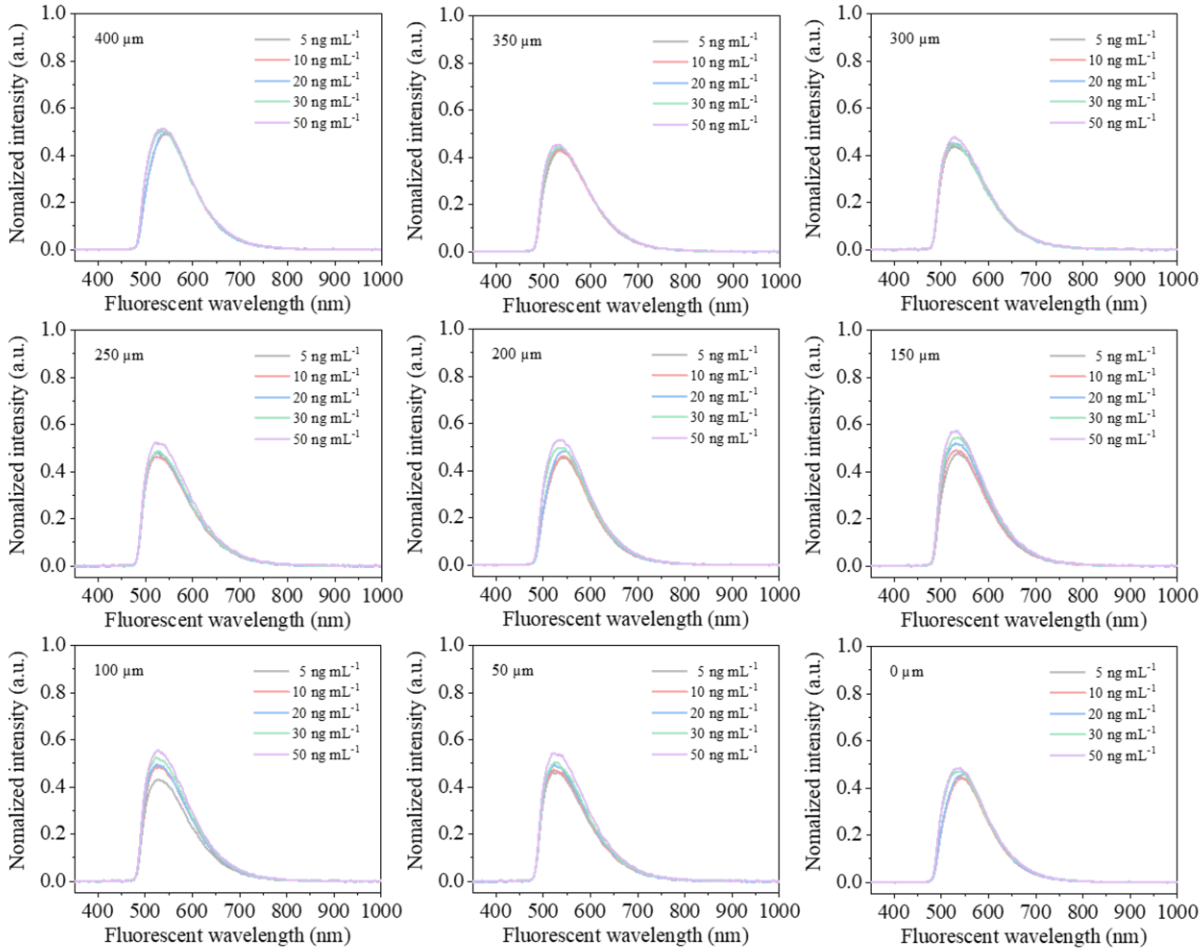

Fig. S3.

Fluorescent intensities of the optical fibers with different end diameters responding to different NTR concentration solutions.

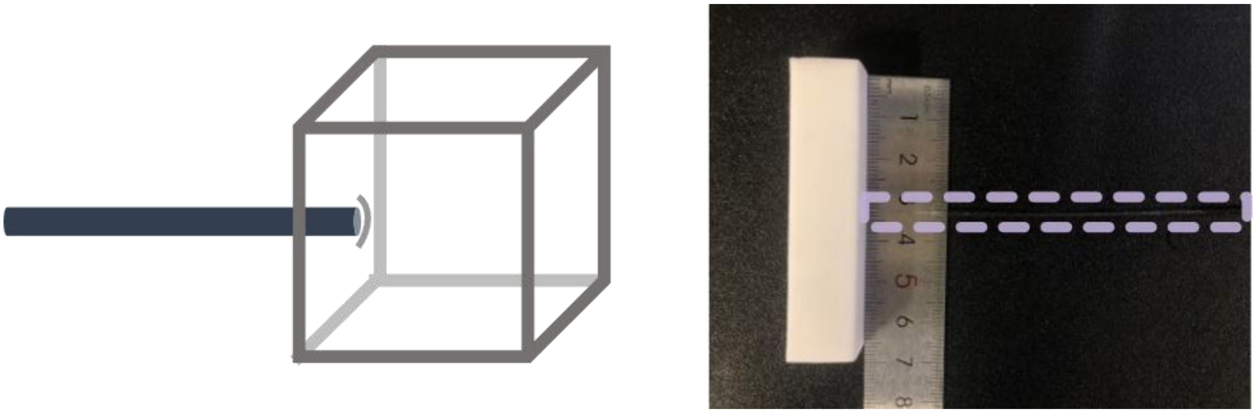

Fig. S4.

Puncturing operation of 400 μm fiber.

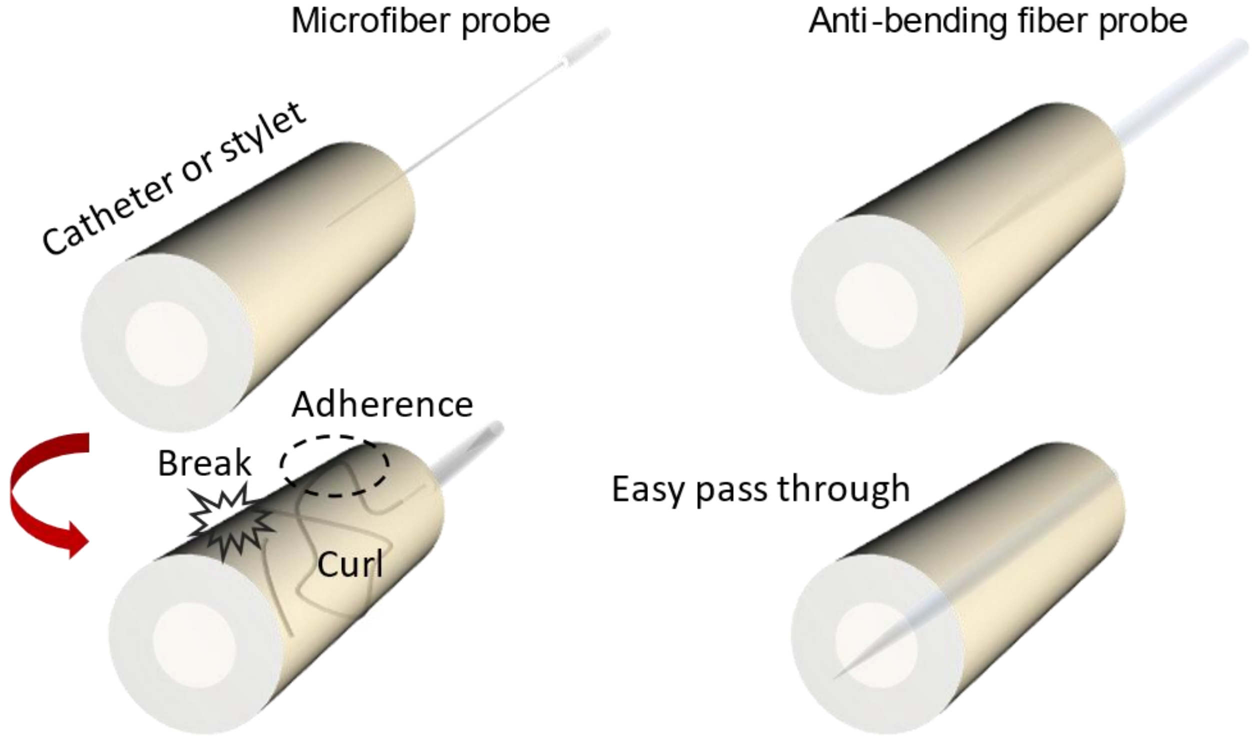

Fig. S5.

Schematics of microfiber probe and anti-bending tapered fiber probe, which are operated in the catheter or sytlet for the interventioal sugery. The highly willowy microfiber structure often suffers from adherence, self-curling, and even breakage in the operatioin, while the anti-bending tapered fiber facilitates the manipulation of probe head in the narrow duct cavity.

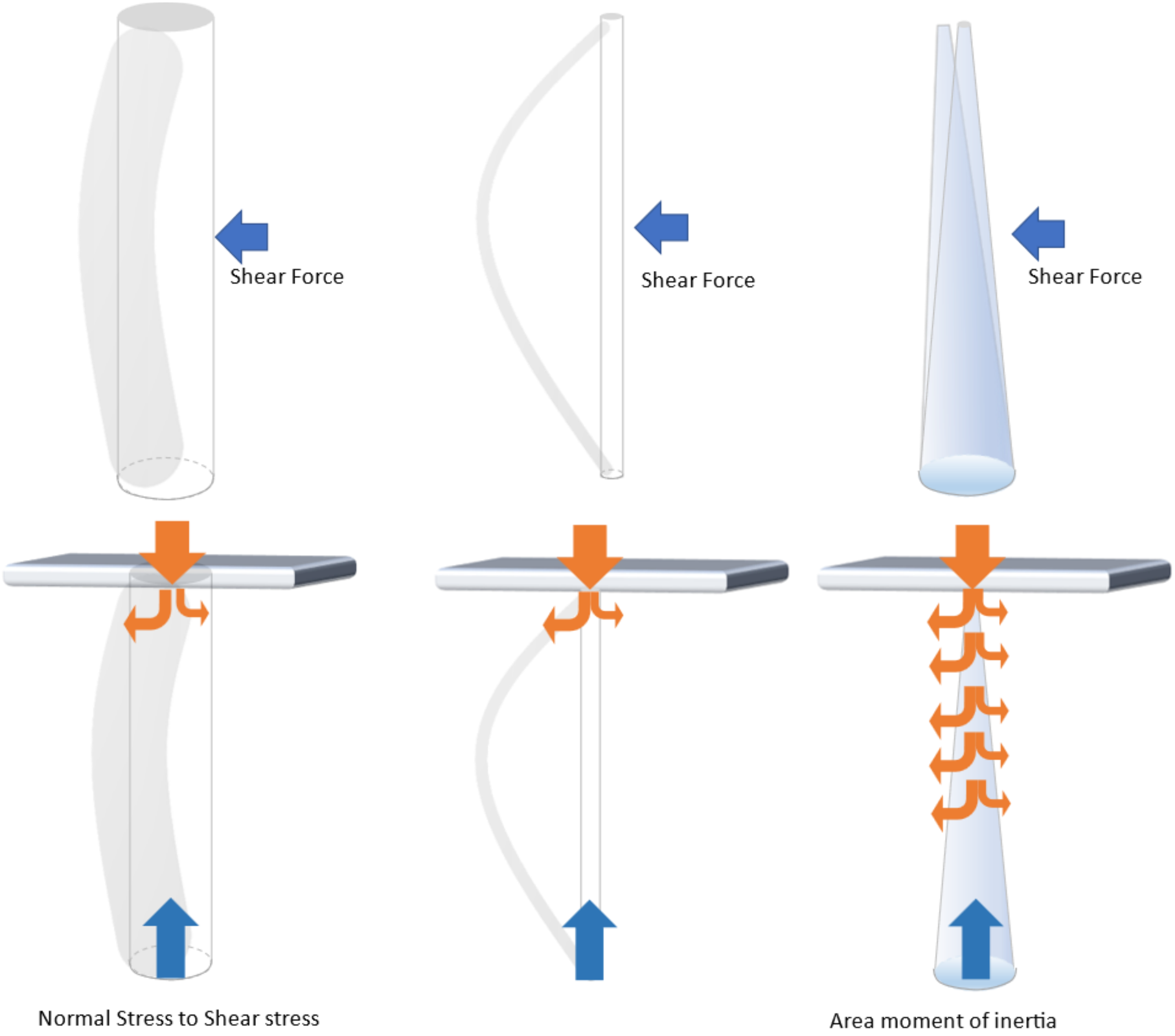

Fig. S6.

The bending characterisics of different fiber head structures in response to ambient stimulation. For the fiber heads with uniform diameter, shear force (derives from air flow,

displacement) will lead to bending of the cylinderical structure, in which the bending amplitude depends on the area inertia moment (sectional resistance moment). Larger diameter confers a larger area inertia moment, which is inversely proportional to the bending amplitude. That is the reason why the thinned microfiber is easy to curl and twine by it self. As the fiber head is exerted to puncture the object, such as tissue or rubber, the shear stress exerting on the object is also inversely proportional to the diameter of the fiber, the thinner fiber head is more likely to stab into the object. However, the counterforce caused by the puncture would be transformed to a similar but more intense shear force on the fiber, leading to break on the uniformly thinned fiber. By contrast, the cone-like fiber head has an excelllent counter-bending ability due to the larger area inertia moment and sectional resistance moment provided by the gradually diameter changed structure.

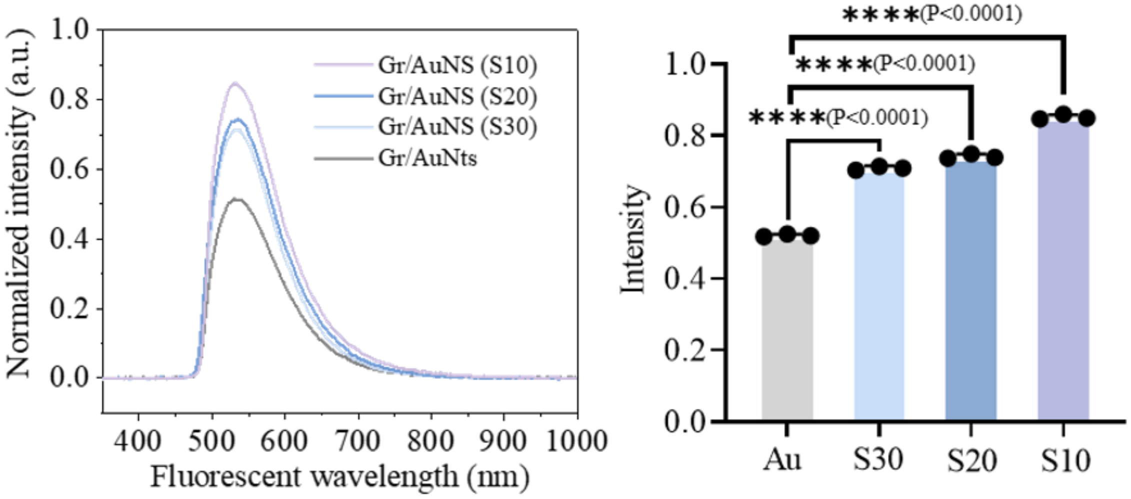

Fig. S7.

The influence of gold nanostars structures on fluorescence sensing in *vitro*. Gr/AuNS solutions with different gold nanostar structures (Gr/Au, Gr/AuNS(S10), Gr/AuNS(S20) and Gr/AuNS(S30)) were tested separately using the fiber sensor. The Gr/AuNS(S10) ($P<0.0001$),

Gr/AuNS(S20) (*P*<0.0001) and Gr/AuNS(S30) (*P*<0.0001) samples exhibited significantly positive results in contrast with the Gr/Au sample.

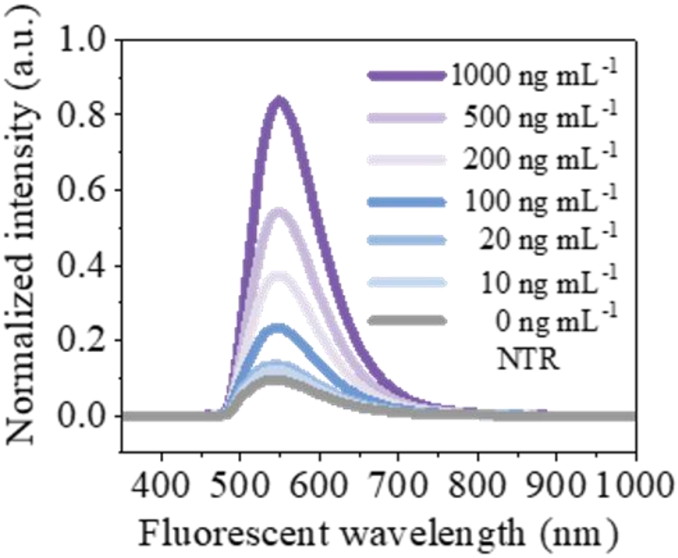

Fig. S8.

The ~550 nm fluorescent signal of Gr/AuNS fiber increases with the increment of the NTR density.

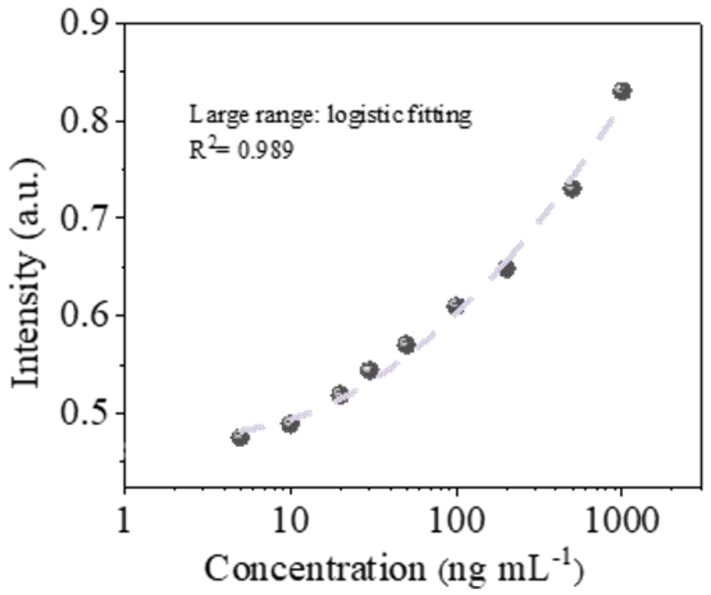

Fig. S9.

The measurement points of signal strength for the wide NTR concentration range. (R^2 = 0.989).

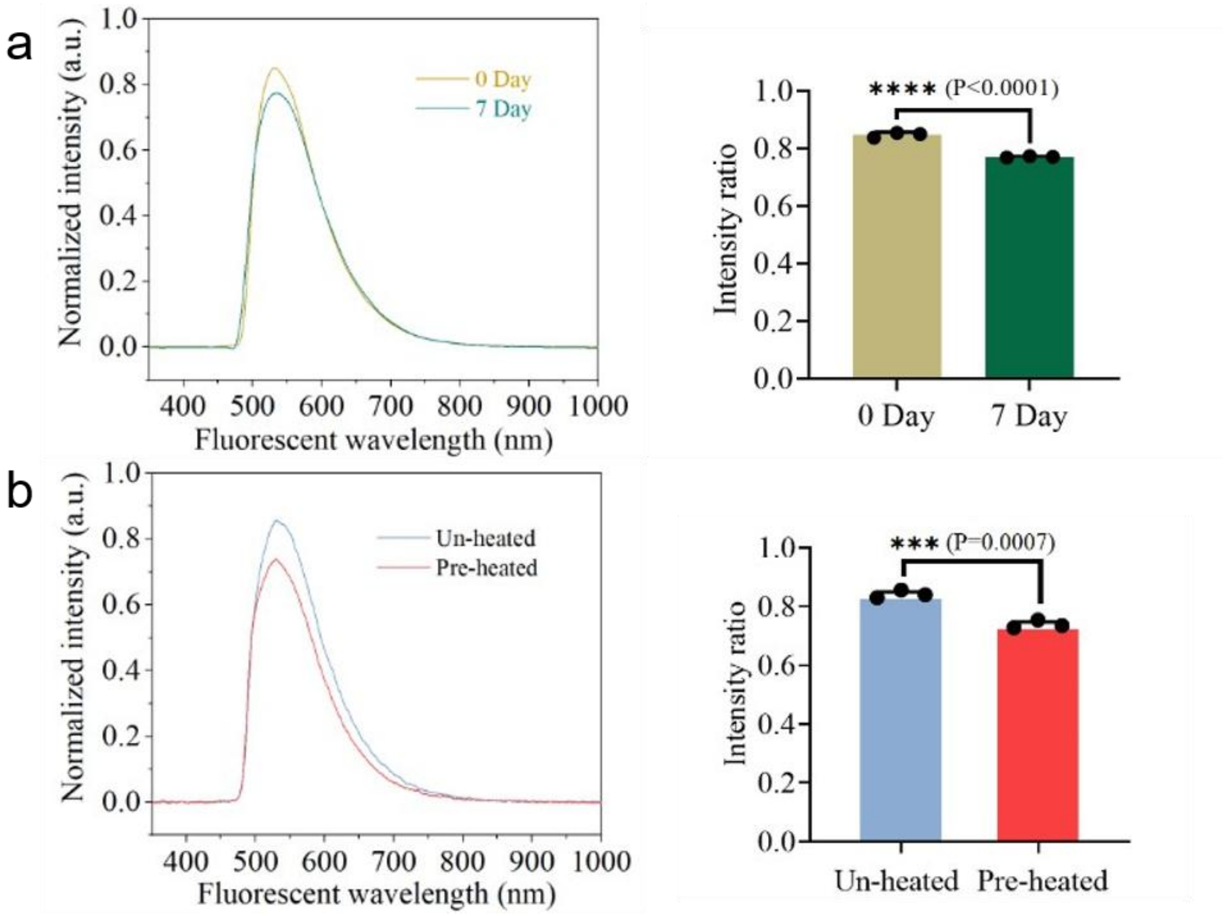

Fig. S10.

Stability testing of sensing. (A) Repeated in *vitro* test using another fiber sensor fabricated by the same functional procedures after 7 days. The comparison of the fluorescent signals between the day 0 fluorescence test and the day 7 fluorescence test. P<0.0001. **(B)** Repeated in *vitro* test using another fiber sensor fabricated by the same functional procedures after heating (~150°C). The comparison of the fluorescent signals between the pre-heating fluorescence test and the post-heating fluorescence test. P=0.0007.

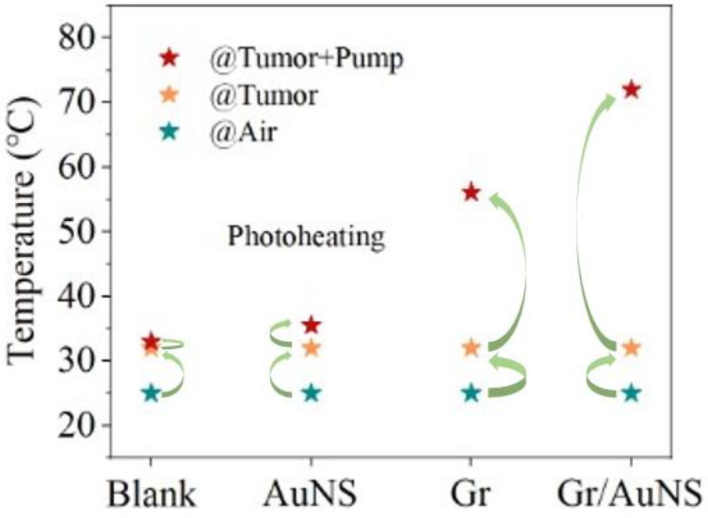

Fig. S11.

The temperature changes logged by the photothermal imager and optical fiber temperature sensor regarding the scenario shift from air to the tumor and then in tumor photoheating. From air to tumor, the fibers were warmed up due to the tumor temperature. By contrast, Gr/AuNS fiber showed the most efficient photothermal conversion as the pump was launched.

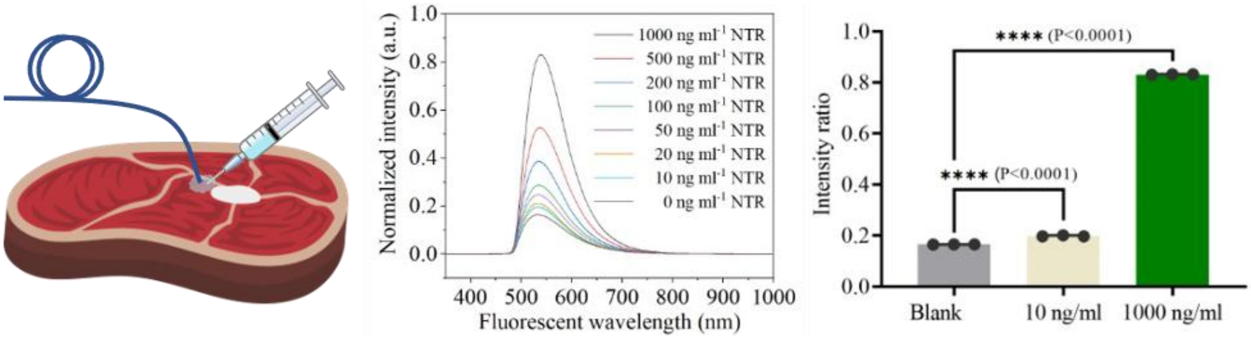

Fig. S12.

***Ex-vivo* sensing.** The NTR and NTR probes mix was injected into the meat tissue after the reaction. And then the fiber was interstitially inserted into the injection spot. The fluorescent signal went up as the increase of NTR density. Both 10 ng mL^{-1} (P<0.0001) and 1000 ng mL^{-1} (P<0.0001) samples exhibited significantly positive results in contrast with the blank sample.

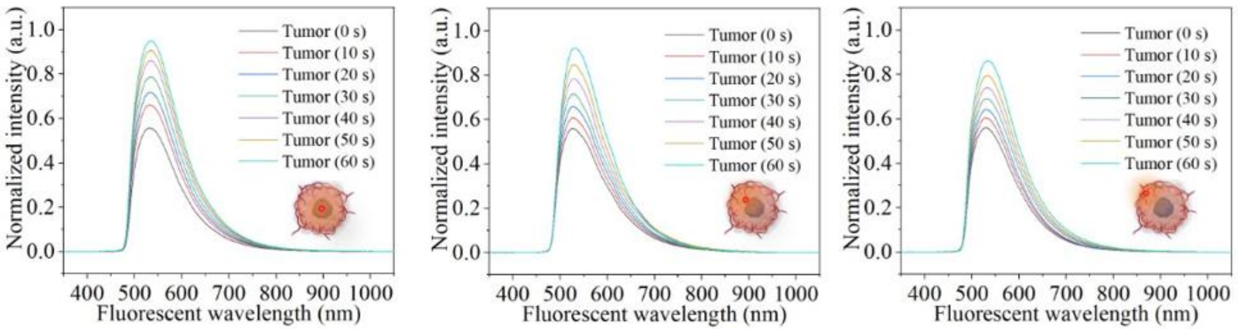

Fig. S13.

Fluorescent signal evolution with time accumulation at the center, off-center, and margin of the tumor. Signal intensity is proportional to tumor density.

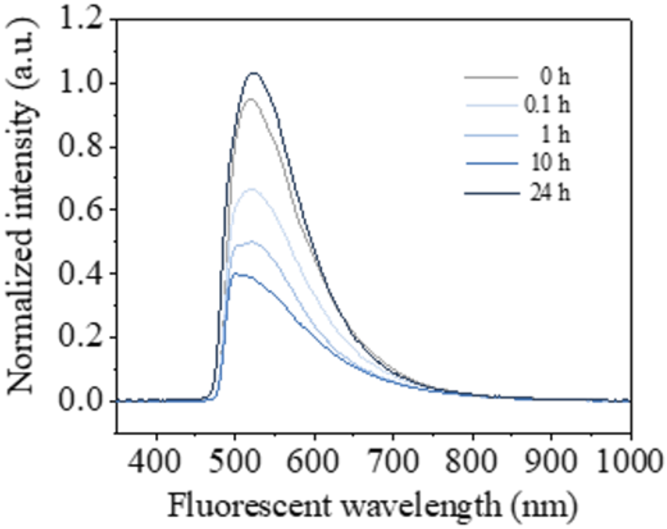

Fig. S14.

Fluorescent signal evolution with time accumulation after PTT of the tumor.

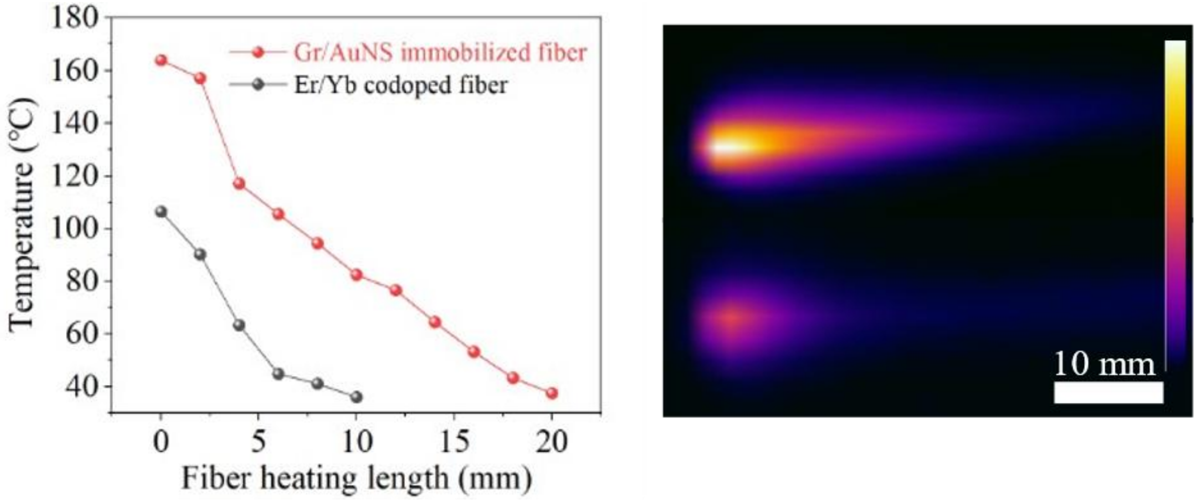

Fig. S15.

Comparison of the thermal action range of Gr/AuNS immobilized fiber and Er/Yb codoped fiber.

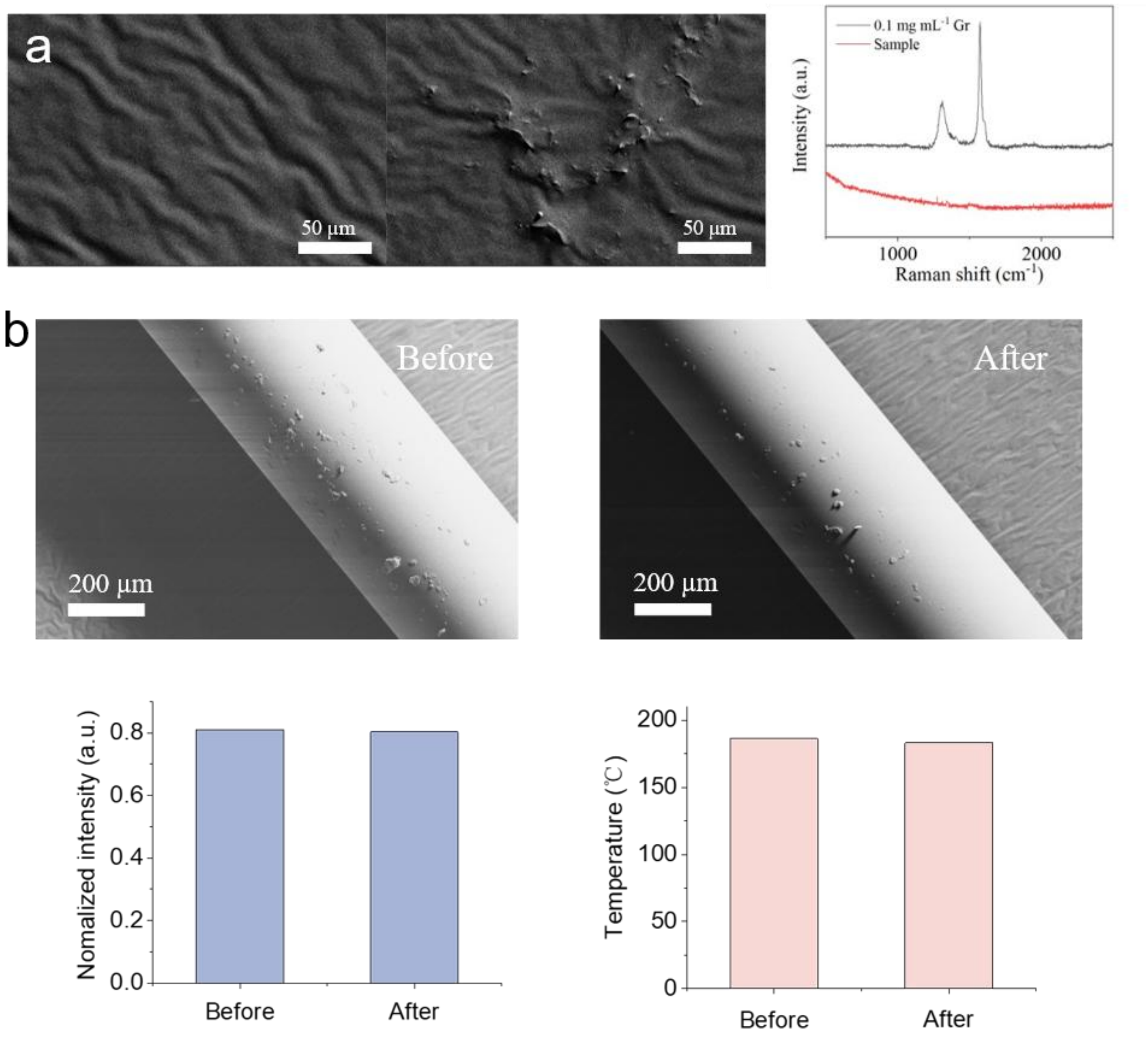

Fig. S16.

Characterization of surface materials on the fiber tip. **(A)** Biosafety was verified by optical fiber probe residual solution characterization. **(**SEM images and Raman spectra). **(B)** Mechanical stability of the functionalizing materials. **(**SEM images, fluorescence efficiency and photothermal temperature).

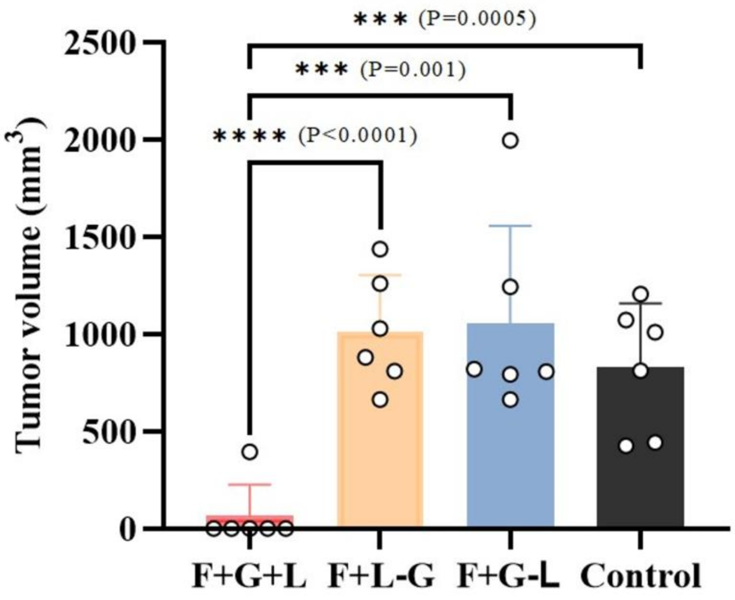

Fig. S17.

Statistical distinctions among the groups at day 30. Statistical analysis is performed by one-way ANOVA followed by Tukey's post hoc test.

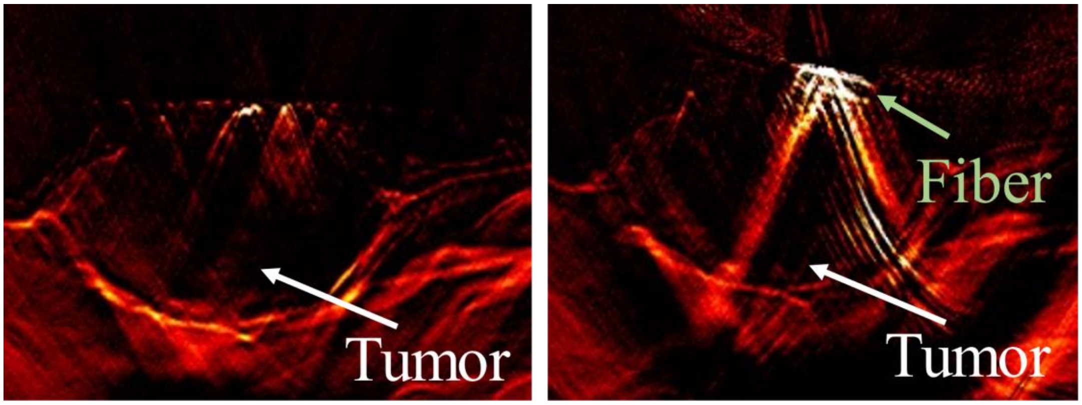

Fig. S18.

Positioning of the fiber probe based on PAI guidance in the tumor of mice.

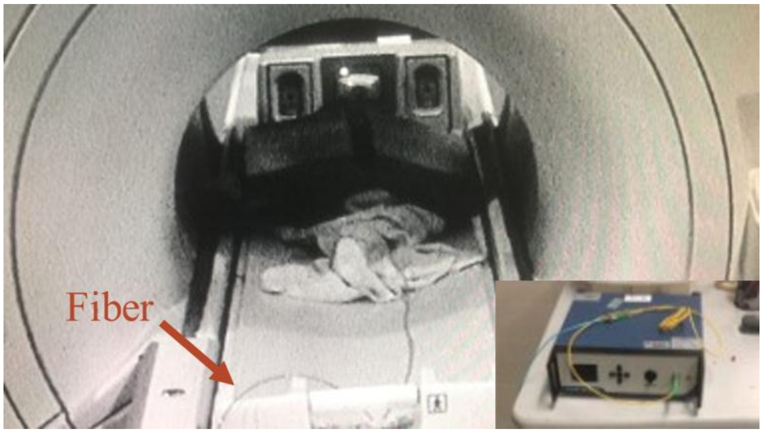

Fig. S19.

Long optical fiber bridges magnetic resonance receiver coil and optoelectronic instruments.

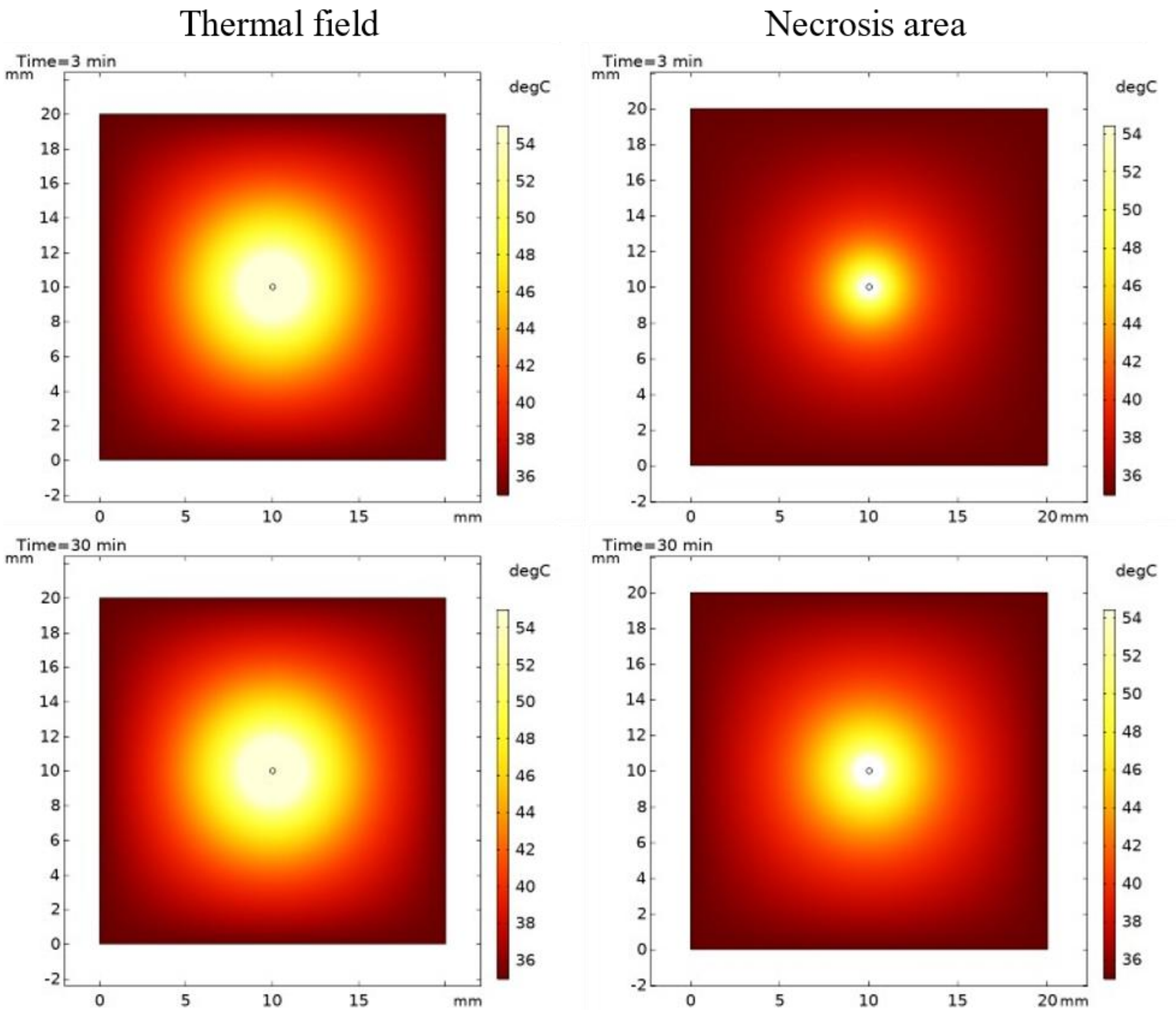

Fig. S20.

Simulation of the fiber treatment effect.

Movie S1.

The monitoring process of iFOT fluorescence sensing demonstration.

Movie S2.

Demonstration of iFOT PTT manipulation and temperature regulation process.

Movie S3.

Operational demonstration of simulated intra-abdominal surgery by iFOT with the assistance of an endoscope.

Movie S4.

Video recording of fiber moving in the tumor of a mouse with ultrasound imaging monitoring.

Movie S5.

Operational demonstration of simulated minimally invasive intervention, diagnosis and treatment by iFOT with model *in vivo*.

Movie S6.

Video recording of fiber moving in the tumor of a mouse with MRI monitoring.

References

1 Hu, D. *et al.* Plasmonic Gold Nanostar Mediated Self-Photothermal Modulation of Microfiber Bragg Grating. *Journal of Lightwave Technology* **40**, 4812-4818 (2022).

2 Ran, Y. *et al.* Fiber-Optic Theranostics (FOT): Interstitial Fiber-Optic Needles for Cancer Sensing and Therapy. *Advanced Science* **9**, 2200456 (2022).

3 Kuila, T. *et al.* Recent advances in graphene-based biosensors. *Biosensors and bioelectronics* **26**, 4637-4648 (2011).

4 Morales-Narváez, E., Baptista-Pires, L., Zamora-Gálvez, A. & Merkoçi, A. Graphene-based biosensors: going simple. *Advanced Materials* **29**, 1604905 (2017).

5 Chaudhary, V. S., Kumar, D., Mishra, G. P., Sharma, S. & Kumar, S. Plasmonic biosensor with gold and titanium dioxide immobilized on photonic crystal fiber for blood composition detection. *IEEE Sensors Journal* **22**, 8474-8481 (2022).

6 Qi, L., Jin, L., Liang, Y., Cheng, L. & Guan, B.-O. Efficiency enhancement of optical tuning for Bragg gratings in rare-earth doped fibers. *IEEE Photonics Technology Letters* **26**, 1188-1191 (2014).

7 Knavel, E. M. & Brace, C. L. Tumor ablation: common modalities and general practices. *Techniques in vascular and interventional radiology* **16**, 192-200 (2013).
